# Identification and characterization of the critical genes encoding Cd-induced enhancement of SOD isozymes activities in Zhe-Maidong (*Ophiopogon japonicus*)

**DOI:** 10.3389/fpls.2024.1355849

**Published:** 2024-03-28

**Authors:** Ruijun Hou, Zhihui Wang, Qian Zhu, Jie Wang, Yifeng Zhou, Ye Li, Huijun Liu, Qian Zhao, Jun Huang

**Affiliations:** ^1^Zhejiang University of Science and Technology, Hangzhou, China; ^2^Key Laboratory of Microbial Technology and Bioinformatics of Zhejiang Province, Hangzhou, China

**Keywords:** *Ophiopogon japonicus*, SOD gene family, cadmium stress, ROS damage, substrate binding sites

## Abstract

Superoxide dismutase (SOD) protects plants from abiotic stress-induced reactive oxygen species (ROS) damage. Here, the effects of cadmium (Cd) exposure on ROS accumulation and SOD isozymes, as well as the identification of significant SOD isozyme genes, were investigated under different Cd stress treatments to Zhe-Maidong (*Ophiopogon japonicus*). The exposure to Cd stress resulted in a notable elevation in the SOD activity in roots. Cu/ZnSODa and Cu/ZnSODb were the most critical SOD isozymes in response to Cd stress, as indicated by the detection results for SOD isozymes. A total of 22 *OjSOD* genes were identified and classified into three subgroups, including 10 *OjCu/ZnSODs*, 6 *OjMnSODs*, and 6 *OjFeSODs*, based on the analysis of conserved motif and phylogenetic tree. *Cu/ZnSOD-15*, *Cu/ZnSOD-18*, *Cu/ZnSOD-20*, and *Cu/ZnSOD-22* were the main genes that control the increase in SOD activity under Cd stress, as revealed via quantitative PCR and transcriptome analysis. Additionally, under various heavy metal stress (Cu^2+^, Fe^2+^, Zn^2+^, Mn^2+^), *Cu/ZnSOD-15*, *Cu/ZnSOD-18*, and *Cu/ZnSOD-22* gene expression were significantly upregulated, indicating that these three genes play a critical part in resisting heavy metal stress. The molecular docking experiments performed on the interaction between oxygen ion (O_2_^•−^) and OjSOD protein have revealed that the critical amino acid residues involved in the binding of Cu/ZnSOD-22 to the substrate were Pro135, Ile136, Ile140, and Arg144. Our findings provide a solid foundation for additional functional investigations on the *OjSOD* genes, as well as suggestions for improving genetic breeding and agricultural management strategies to increase Cd resistance in *O. japonicus*.

## 1 Introduction

Over the past few years, there has been a noticeable rise in Cd contamination within ecosystems, primarily attributed to anthropogenic activities such as non-ferrous metal smelting, cement production, and the application of fertilizers. A previous study reported a discernible level of Cd contamination across China, noting that the pollution was more pronounced in southern regions than in northern areas (Yao et al., 2022). Under conditions of extremely low concentrations of Cd stress, plants may also exhibit hazardous consequences. For instance, Cd stress can cause yellowing of plant root tips, root shrinkage, and dwarfing of seedlings, thereby impeding overall plant development and causing a notable decline in the yield of medicinal plants (Fan et al., 2023). The occurrence of Cd toxicity in plants is commonly linked to the interference of diverse metabolic processes involving the absorption and distribution of water and nutrients, photosynthesis, and redox reactions (Zhang et al., 2020). Cd ions have been reported to combine with enzyme active centers or protein sulfhydryl groups in plants, replacing the necessary metal elements iron (Fe), zinc (Zn), or calcium (Ca), releasing free radical ions and causing oxidative stress reactions, eventually leading to cell membrane lipid peroxidation and cell membrane damage (Yang et al., 2005).

Zhe-Maidong, scientifically known as a species of *O. japonicus* widely planted in Zhejiang province, is a traditional Chinese herbal medicine (Liu et al., 2017). Numerous contemporary pharmacological investigations have substantiated the abundance of flavonoids, saponins, and polysaccharides in Zhe-Maidong, thereby establishing its advantageous properties in terms of antioxidation, cardiovascular safeguarding, anti-inflammatory response, anticancer activity, and immune system regulation (Chen et al., 2022). Zhe-Maidong has been extensively cultivated in Zhejiang Province of southern regions of China for over 2 millennia, and it is recognized for its exceptional quality, surpassing that of all other *O. japonicus* cultivars (Chen et al., 2022; Jiang et al., 2022; Zhao et al., 2022). However, previous studies have indicated that the incidence of Cd exceeding the permissible limits in agricultural soil samples obtained from Zhejiang Province between 2016 to 2020 approaches a substantial 12.06% (Xiang et al., 2022). In addition, it was found that several batches of *O. japonicus* samples displayed elevated levels of Cd (Zhao et al., 2022). The presence of Cd stress has emerged as a significant constraint on the growth and development of Zhe-Maidong in the Zhejiang region.

Plants possess a range of antioxidant mechanisms, which consist of both enzymatic and non-enzymatic defense systems, helping plants to withstand the harmful effects of Cd stress (Gill et al., 2015). The enzymatic antioxidant defense system is composed of superoxide dismutase (SOD), catalase (CAT), ascorbate peroxidase (APX), and peroxidase (POD) (Fujita and Hasanuzzaman, 2022). SOD is a crucial enzyme that serves as the initial defense mechanism in the antioxidant defense system of plants (Sen Raychaudhuri and Deng, 2000). Its primary role is to dismutate superoxide radicals into hydrogen peroxide and oxygen through a process known as disproportionation (Gill and Tuteja, 2010; Gill et al., 2015). According to Che et al., the application of cold stress treatment resulted in a significant increase in the overall activities of SOD, POD, and CAT in potato plants (Che et al., 2020). Conversely, Bhuiyan et al. found that drought stress treatment elevated APX and CAT activities in *Brassica rapa* L (Bhuiyan et al., 2019). Prior studies have extensively investigated the reaction of antioxidant enzymes when subjected to Cd stresses, revealing that various plant species exhibit distinct responses regarding their antioxidant enzyme activity. Zaid et al. observed that exposure to Cd stress in *Mentha arvensis* L. resulted in an upregulation of the antioxidant enzymes SOD and CAT (Zaid et al., 2020). According to Ahanger et al., the exposure of *Vigna angularis* to Cd stress resulted in an increase in the activities of SOD and APX, whereas the activity of CAT was seen to decrease (Ahanger et al., 2020). In the study by Al Mahmud et al., the presence of mild and severe Cd stress in *Brassica juncea* L. resulted in an elevation of SOD activity, whereas the CAT activity declined as the intensity of the stress grew (Al Mahmud et al., 2018). Additionally, Cd treatment resulted in a considerable rise in plant leaf SOD activity in muskmelon, which tended to decrease as Cd content increased (Wang et al., 2022).

The family of genes encoding SOD isoenzymes has been identified in a variety of plants, including *Nicotiana tabacum* (Huo et al., 2022), *Salvia miltiorrhiza* (Han et al., 2020), soybeans (Aleem et al., 2022), *Daucus carota* (Zameer et al., 2022), potato (Rudić et al., 2022), wheat (Tounsi et al., 2023), barley (Zhang et al., 2021b), oilseed rape (Su et al., 2021), watermelon, and melon (Zhang et al., 2021a) and others. Based on the bound metal cofactor, SODs can be categorized into three classes: Cu/ZnSOD, FeSOD, and MnSOD (Li et al., 2021). SOD isozymes in plants have been demonstrated to perform essential roles in their response to a wide range of adverse environmental conditions. Previous findings indicated that the Cu/ZnSOD isozymes present in *Kandelia obovata* root tissues play a more significant role in enhancing the plant’s tolerance to Cd stress compared to the FeSOD isozyme (Pan et al., 2020). FeSOD and Cu/ZnSOD expression levels were comparatively high in rice salt-tolerant varieties, whereas MnSOD expression levels were more significant in sensitive varieties (Kaur et al., 2016). Another study in *Cicer arietinum* L. reveals that MnSOD, instead of FeSOD and Cu/ZnSOD, contributed to the Cd-induced increase in SOD activity (Sakouhi et al., 2021). However, the effect of Cd stresses on the oxidative homeostasis in Zhe-Maidong and the corresponding response of antioxidant enzymes, particularly SOD, have yet to be investigated.

In this study, the Zhe-Maidong cultivar was used to explore the effect of varying Cd concentrations on the physiological traits related to redox reactions under hydroponic and soil culture conditions, including the contents of O_2_^•−^, hydrogen peroxide (H_2_O_2_), and malondialdehyde (MDA), as well as the activities of antioxidant enzymes. Transcriptomic data were further used to identify and evolutionary analyze the gene family encoding the SOD enzyme, which increased significantly in response to Cd stress, in order to determine critical SOD genes that control SOD activity. Based on the findings, the expression patterns of the predominant SOD genotypes were detected in response to various heavy metal stresses. The 3D structure of the SOD family proteins was constructed using the homology modeling technique, and the molecular docking technique was further applied to investigate its binding state with the substrate superoxide anion to resolve the key active sites of the SOD family members. Our findings contribute to the comprehension of the possible interplay between Cd stress-induced ROS and SOD isozymes activity, as well as the functions of candidate *OjSOD* genes in Cd stress responses of Zhe-Maidong.

## 2 Materials and methods

### 2.1 Plant materials and abiotic stress treatments

Zhe-Maidong (*O. japonicus* in Zhejiang) seedlings were selected from Cixi, Zhejiang Province when they were one year old. The plants were then exposed to a variety of Cd^2+^ stressors. The management of Cd stress in hydroponics and soil culture follows below.

**Experiment I**: Three Cd stress treatments, Cd-0 (0.14 mg/kg), Cd-1 (0.35 mg/kg), and Cd-2 (0.61 mg/kg), were established utilizing soil culture. The Cd content of Cd-1 exceeds the national Grade II value for soil Cd levels in China (0.3 mg/kg), which can be regarded as Cd stress treatment. Each treatment was replicated in 5 pots, with 2 plants per pot. The pots were placed in a greenhouse with natural light conditions, with an average daily temperature of 25°C to 28°C. The plants were managed uniformly and irrigated every 2-3 days as required. Samples were taken after 90 days of plant growth.

**Experiment II**: For hydroponics culture, the experimental conditions consisted of three Cd treatment groups: control (referred to as Cd-0), 1 mg/L (referred to as Cd-M, the maximum permitted Cd content in soil for agriculture), and 10 mg/L (referred to as Cd-H) (Zhao et al., 2022). The nutrient solution was replaced every three days. Following a treatment period of 30 days in the artificial climate chamber, samples of the roots were obtained and promptly frozen in liquid nitrogen before being stored at -80°C.

**Experiment III:** Multiple heavy metal stress investigations were performed on Zhe-Maidong to analyze the effects of heavy metal stresses on *SOD* gene expression using Cu^2+^, Fe^2+^, Zn^2+^, and Mn^2+^. Each metal stress consisted of two different concentrations: Cu-L (10 mg/Kg), Cu-H (100 mg/Kg), Fe-L (2 mg/Kg), Fe-H (20 mg/Kg), Mn-L (10 mg/Kg), Mn-H (100 mg/Kg), Zn-L (25 mg/Kg), Zn-H (250 mg/Kg). The amounts of Cu-H, Fe-H, Mn-H, and Zn-H were set according to the threshold for potential soil pollution in agricultural land in China. Standardized plant management and irrigation every 1-2 days were conducted. After six days of treatment, leaf and root samples were stored at -80°C.

### 2.2 Detection of ROS accumulation, SOD isozymes, and native-PAGE identification of SOD isozymes

The concentrations of O_2_^•−^, H_2_O_2_, MDA, and SOD activity were determined using the corresponding assay kits (Sangon Biotech, Shanghai, China). CAT, APX, and POD activities were determined according to the protocols described in previous studies (Hasanuzzaman and Fujita, 2011; Ozgur et al., 2014). Three biological replicates were used in each experiment. The Histochemical staining of O_2_^•−^, level in Zhe-Maidong after Cd stress was based on the method previously described in the literature (Zhang et al., 2011). The Native-PAGE/activity staining of SOD was conducted following the previously described methods (Ozgur et al., 2014; Zhao et al., 2018).

### 2.3 Identification and characterization of the SOD gene family

BlastP (protein blast) and the Hidden Markov Model (HMM) were both used to identify *SOD* genes in the Zhe-Maidong transcriptome data obtained from previous study (https://ngdc.cncb.ac.cn/gsa/) (GSA: CRA008254). BlastP database was constructed using nine *Arabidopsis thaliana* SOD protein sequences (AT5G18100.1/AtCu/ZnSOD-1, AT1G08830.1/AtCu/ZnSOD-2, AT1G12520.1/AtCu/ZnSOD-3, AT2G28190.1/AtCu/ZnSOD-4, AT3G10920.1/AtMnSOD-5, AT3G56350.1/AtMnSOD-6, AT4G25100.1/AtFeSOD-7, AT5G51100.1/AtFeSOD-8, AT5G23310.1/AtFeSOD-9) as query sequences. The e-value was set to 1e-10 for querying, while filtering was carried out based on the identity value. The protein sequences of nine AtSODs were acquired from TAIR (https://www.arabidopsis.org/).

The HMM search tool of HMMER on Linux was also used to search for predicted OjSOD proteins. The HMM files of SOD_Cu (PF00080), SOD_Fe_N (PF00081), and SOD_Fe_C (PF02777) were obtained from the Pfam web (http://pfam.xfam.org/). Then, all obtained protein sequences were used to verify the presence of the *SOD* domain by SMART (http://smart.embl-heidelberg.de/) and NCBI-CDD (http://www.ncbi.nlm.nih.gov/structure/cdd/wrpsb.cgi) for identifying the SOD proteins in Zhe-Maidong. The *SOD* genes in other plant species, including *Asparagus officinalis*, *Panax notoginseng*, and *Panax ginseng*, were identified using the same methods, and the genomes of these species were downloaded from the Phytozome 13 (https://phytozome-next.jgi.doe.gov/) and the National Center for Genome Sciences Data (https://ngdc.cncb.ac.cn/gwh/). The *SOD* genes of *A. thaliana* and *Oryza sativa* were obtained from the previous study (Yadav et al., 2019).

The physicochemical characteristics of OjSOD proteins were calculated using ExPASY (https://web.expasy.org/protparam), and the subcellular location of these proteins was predicted using Cell-PLoc2.0 (http://www.csbio.sjtu.edu.cn/bioinf/Cell-PLoc-2/).

### 2.4 Analysis of phylogenetic, structural domain, conserved motifs, and GO enrichment

The SOD protein sequences were aligned using the MUSCLE server, model selection was performed using IQ-TREE (Nguyen et al., 2015), and an ML phylogenetic tree was constructed using 86 SOD protein sequences from *A. thaliana*, *O. sativa*, *A. officinalis*, *P. notoginseng*, and *P. ginseng*. Evolview (http://evolgenius.info/#/) was used to beautify the evolutionary tree. The prediction of the structural domain was analyzed using PFAM (http://pfam.xfam.org/search). Conserved motif analysis of the *OjSOD* genes was performed using the MEME Suite server. Here, we set the motif numbers as 9, the minimum length of the motif to 8, and the maximum length to 200. Gene ontology annotations were obtained through blast2go in the NCBI non-redundant protein sequences database (NR) (http://www.ncbi.nlm.nih.gov/) top 10 annotation results and combined with the Pfam data from Pfam (Protein family) database from the InterPro (https://www.ebi.ac.uk/interpro/) scan at the same time.

### 2.5 Prediction of the 3D structure and molecular docking

The SWISS-MODEL server predicted the three-dimensional structures of OjSOD proteins (Waterhouse et al., 2018). The PyMOL program (http://www.pymol.org) was used to visualize 3D structures. For molecular docking, first, we downloaded ligands (O_2_^•−^ and H_2_O_2_) files on PubChem database (https://pubchem.ncbi.nlm.nih.gov/). Next, the preparation of ligands and SOD proteins was carried out according to the previous method (Bhardwaj et al., 2019). Then, the O_2_^•−^ and H_2_O_2_ ligands files were docked to the SOD proteins by the CDOCKER program of Discovery Studio. The top 10 poses were preserved with the stable binding modes. The binding mode with the highest -CDocker Energy score was chosen to identify the binding site.

### 2.6 Analysis of the expression of *OjSOD* genes by transcriptome data

RNA-Seq data of *OjSOD* genes under different concentrations of Cd stress was downloaded from GSA (https://ngdc.cncb.ac.cn/gsa). A heat map was generated using FPKM values (Log_2_(FPKM + 1)) for *OjSOD* genes, and the expression of all *OjSOD* genes was normalized by rows. Based on the generated heat map, genes showing significant changes were selected for further analysis.

### 2.7 RNA extraction, cDNA preparation, and quantitative real-time PCR analysis

RNA extraction and cDNA preparation method for Zhe-Maidong were performed as described in previous literature (Ueda et al., 2013). Real-time quantitative PCR reactions were performed using iTaq Universal SYBR Green Supermix (Bio-Rad, USA). The PCR reactions were conducted on a Bio-Rad CFX96 Real-Time System (Bio-Rad, USA) following the manufacturer’s instructions. The *RPL35* gene (*Cluster_21637.81227*) was chosen as the internal reference gene for standardization. Expression levels of various *OjSOD* genes were analyzed using the 2^-ΔΔCT^ method (Livak and Schmittgen, 2001). All primers used in this research were listed in **Supplementary Table S1**. Mean values and standard errors were derived from three distinct biological replicates.

### 2.8 Data analysis

Data were analyzed using SPSS (Chicago, USA). Differences in means were compared by the Least Significant Difference (LSD) method, with different letters indicating significant differences (p < 0.05). The data were presented in the form of the means of three biological repetitions ± standard deviation (SD) in the figures.

## 3 Results

### 3.1 Effect of Cd exposure on ROS accumulation and antioxidant enzyme activities in Zhe-Maidong

**Figure 1:**
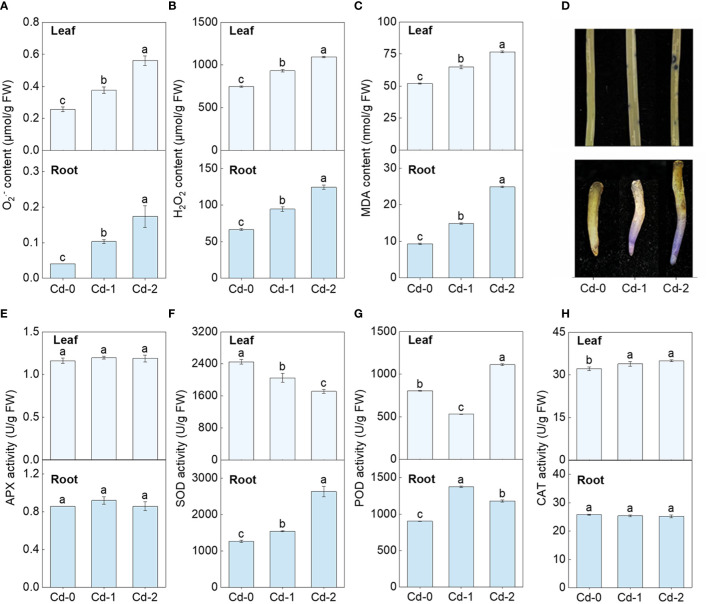
Effect of Cd exposure on ROS accumulation and antioxidant enzyme activities in different tissues of Zhe-Maidong. **(A-C)** Differences in O_2_^•−^, H_2_O_2_, and MDA content among various Cd exposure. **(D)** Histochemical localization of O_2_^•−^ production by NBT staining. **(E-H)** Analysis of SOD, POD, CAT, and APX activity in Zhe-Maidong exposed to Cd stresses. Cd-0, Cd-1, and Cd-2 indicated 0.14 mg/kg Cd, 0.35 mg/kg Cd, and 0.61 mg/kg Cd treatments by soil cultivation. Vertical bars represent standard deviation (SD) from three biological replicates. Different letters indicated significant differences in the LSD test with p <0.05.

**Figure 2:**
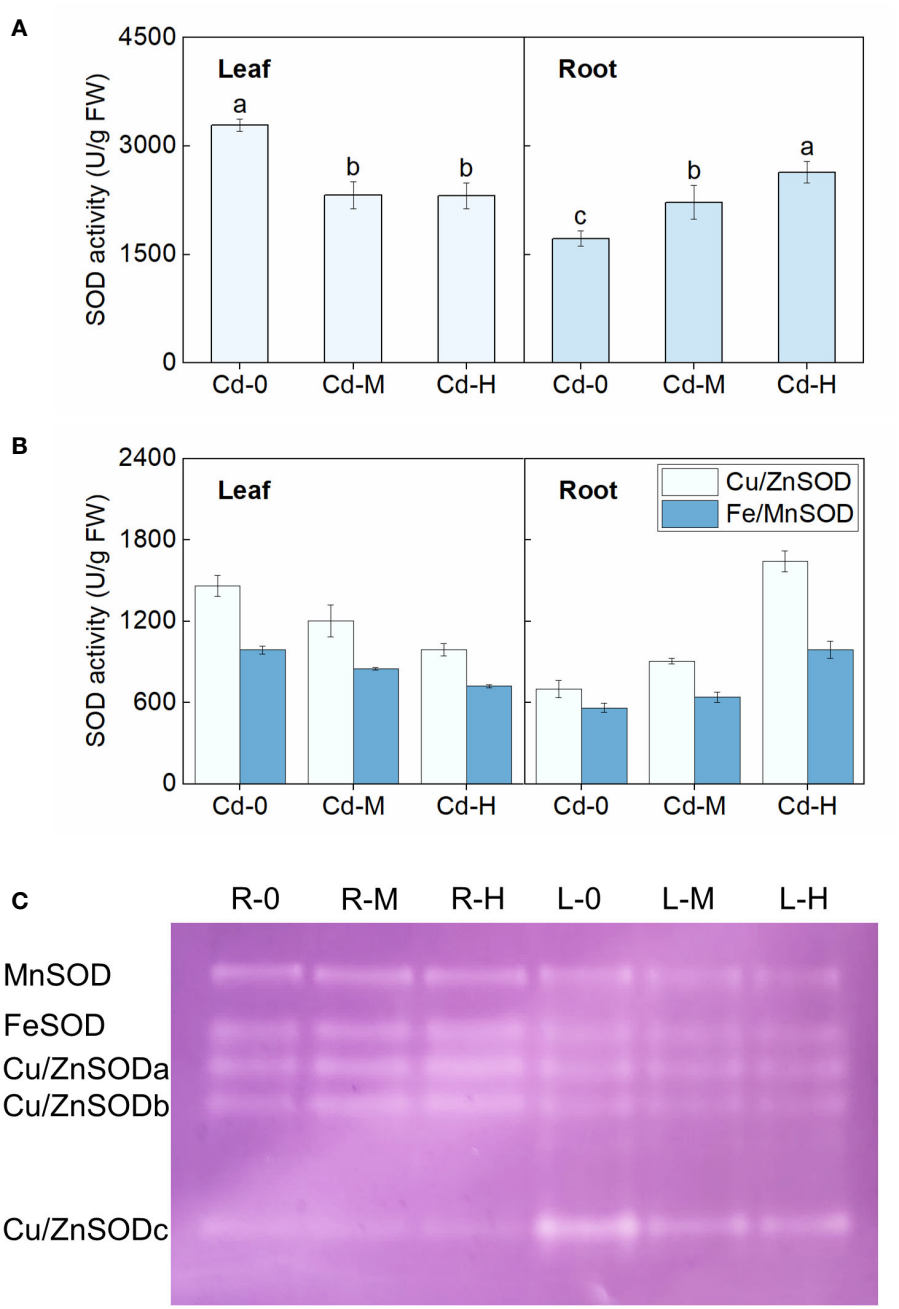
Effect of Cd exposure on SOD activity in different tissues of Zhe-Maidong. **(A, B)** Analysis of SOD, Fe/MnSOD, and Cu/ZnSOD activity in Zhe-Maidong exposed to various Cd stresses. Cd-0, Cd-M, and Cd-H indicated control, 1 mg/L Cd, and 10 mg/L Cd treatments by hydroponics. **(C)** Various SOD isozymes in response to Cd exposure in different tissues. Equal amounts of protein were subjected to Native-PAGE for each lane to identify different isozymes for SOD. R and L represented root and leaf, respectively. 0, M, and H indicated Cd-0, Cd-M, and Cd-H treatments. Vertical bars represent standard deviation (SD) from three biological replicates. Different letters indicated significant differences in the LSD test with p <0.05.

Upon exposure to Cd stress, there was a significant increase in the contents of O_2_^•−^, H_2_O_2,_ and MDA in both the leaves and roots of Zhe-Maidong (**Figures 1A-C**). Additionally, the extent of this increase was directly correlated to the severity of the Cd stress. The NBT staining also indicated that O_2_^•−^ accumulation in leaves and roots increased markedly under Cd exposure, which coincides well with the results obtained from the chemical analysis (**Figures 1D, H**). These findings provide evidence supporting the occurrence of oxidative damage induced by Cd stress in Zhe-Maidong. The activity of antioxidant enzymes was further examined in Zhe-Maidong roots and leaves subjected to Cd stress (**Figures 1E–H**), with the results revealing significant diversity in the responses of several antioxidant enzymes to Cd exposure in different tissues. APX activity in both roots and leaves was significantly elevated under medium and high Cd treatments (Cd-M and Cd-H) compared to the control. CAT activity in leaves increased greatly under Cd-H but remained stable in roots. Interestingly, SOD and POD activity in leaves and roots responded entirely differently to various Cd stresses. For example, SOD activity in roots increases when Cd exposure increases, and vice versa in leaves. Furthermore, it was shown that SOD activity in roots significantly increased by 2.08 times compared to the control group when subjected to severe Cd stress (Cd-H). The observed increase in enzyme activity was more prominently observed in SOD in comparison to APX and POD. On this basis, we also examined the response of SOD activity to Cd stress in the roots and leaves of Zhe-Maidong under hydroponic conditions. The results were consistent with the results obtained from soil culture (**Figure 2A**), indicating that SOD plays a pivotal role in alleviating the adverse effects of Cd-induced ROS damage in the roots.

The activities of two SOD isoenzymes, Cu/ZnSOD and Fe/MnSOD, were further examined under different Cd stress treatments, respectively (**Figure 2B**). Results showed that Cu/ZnSOD activity was significantly higher than that of Fe/MnSOD both in leaves and roots. The observed variations in Cu/ZnSOD and Fe/MnSOD enzyme activities in both roots and leaves under different Cd stress treatments appeared to be consistent with the overall response of total enzyme activity to Cd stress. For example, the activities of both Cu/ZnSOD and Fe/MnSOD in roots increased significantly as Cd stress intensified, whereas the opposite was true in leaves. Cu/ZnSOD activity in the roots increased by 28.84% and 133.78%, respectively, when exposed to Cd-M and Cd-H. These values were found to be considerably greater than the Fe/MnSOD activity, which increased by 14.23% and 76.59%, respectively, under the same stress conditions. Native-PAGE staining was then utilized to detect SOD isoenzymes in roots and leaves exposed to various Cd stress treatments (Ozgur et al., 2014). According to the findings of previous studies, the bands were assigned the names MnSOD, FeSOD, Cu/ZnSODa, Cu/ZnSODb, and Cu/ZnSODc in descending order (**Figure 2C**). Significant variations in the reaction of SOD isoenzymes to Cd stress can be observed in different organs of Zhe-Maidong. Native-PAGE staining showed that the amount of MnSOD, FeSOD, and Cu/ZnSOD in leaves exhibited a consistent decrease in response to various Cd stress conditions. MnSOD enzyme activity in roots remained relatively constant, and FeSOD, Cu/ZnSODa, and Cu/ZnSODb enzyme activities all rose dramatically, while Cu/ZnSODc enzyme activity decreased significantly under Cd stress (**Figure 2C**). These findings indicated that Cu/ZnSODa and Cu/ZnSODb were the most dominant isoenzymes of SOD in response to Cd stress.

### 3.2 Identification, phylogenetic analysis, and GO enrichment analysis of the *OjSOD* gene family in the Zhe-Maidong

A total of twenty-two *OjSOD* genes were identified from Zhe-Maidong by performing HMM search and BlastP analysis (**Supplementary Table S2**). The nomenclature of these genes was determined based on the classification used for homologous SOD genes in well-established species, such as *A. thaliana* and *O. sativa*. SOD from Zhe-Maidong was classified into three subfamilies, Cu/ZnSOD, FeSOD, and MnSOD. The OjSOD proteins exhibited a diversity of lengths, ranging from 83 to 303 amino acids (aa), with an average length of 168 amino acids, and their molecular weights varied from 8.67 kDa to 33.94 kDa. Almost all members of OjSODs were found to be acidic, with predicted isoelectric point (pI) values less than 7. Aliphatic index (AI) analysis revealed that the OjCu/ZnSOD proteins were more resistant to heat than the OjFeSOD proteins, but both were less heat-resistant than the OjMnSOD proteins. The hydrophilicity assessment, known as GRAVY, indicated that OjSOD proteins exhibited hydrophilic properties (**Table 1**). Predictions of the subcellular location showed that OjMnSOD and OjFeSOD proteins were primarily localized in mitochondria; however, some OjFeSOD proteins were localized in chloroplasts, implying that these *OjFeSOD* genes may be involved in the scavenging of photosynthetically produced ROS.

**Table 1:** Physicochemical characteristics and subcellular location of OjSOD proteins.

GeneID	Gene name	Length	MolWt	pI	Aliphatic index	GRAVY	Cell-Ploc
*Cluster-4747.0*	*OjMnSOD-1*	216	24284.34	6.54	89.35	-0.356	Mitochondrion.
*Cluster-21637.76074*	*OjMnSOD-2*	130	14447.74	9.7	90.85	-0.131	Mitochondrion.
*Cluster-28452.0*	*OjMnSOD-3*	115	13377.06	5.47	88.17	-0.243	Mitochondrion.
*Cluster-19202.0*	*OjMnSOD-4*	128	14601.77	6.71	97.58	-0.111	Mitochondrion.
*Cluster-28452.1*	*OjMnSOD-5*	143	16107.48	6.13	93.57	-0.173	Mitochondrion.
*Cluster-21637.76074.1*	*OjMnSOD-6*	118	13190.94	5.06	88.47	-0.384	Mitochondrion.
*Cluster-21168.0*	*OjFeSOD-7*	132	15282.48	9.57	73.86	-0.687	Mitochondrion.
*Cluster-21637.17725*	*OjFeSOD-8*	265	30495.83	7.25	81.32	-0.384	Mitochondrion.
*Cluster-21637.89309*	*OjFeSOD-9*	303	33944.87	5.51	72.48	-0.551	Chloroplast.
*Cluster-11491.0*	*OjFeSOD-10*	207	22942.39	5.83	69.9	-0.387	Mitochondrion.
*Cluster-21637.825*	*OjFeSOD-11*	189	21385.09	6.66	71.8	-0.471	Mitochondrion.
*Cluster-25607.0*	*OjFeSOD-12*	196	22860.78	6.47	70.61	-0.587	Mitochondrion.
*Cluster-10048.0*	*OjCu/ZnSOD-13*	218	23071.65	6.41	84.08	-0.043	Chloroplast.
*Cluster-27087.0*	*OjCu/ZnSOD-14*	158	16397.08	6.04	74.05	-0.527	Chloroplast.Cytoplasm.
*Cluster-7054.0*	*OjCu/ZnSOD-15*	169	17724.52	6.46	68.05	-0.56	Cytoplasm.Mitochondrion.
*Cluster-5811.0*	*OjCu/ZnSOD-16*	109	11058.08	6.21	67.06	-0.484	Chloroplast.Cytoplasm.Mitochondrion
*Cluster-10466.0*	*OjCu/ZnSOD-17*	135	13725.18	5.81	88.15	-0.164	Chloroplast.
*Cluster-21637.67994*	*OjCu/ZnSOD-18*	83	8672.78	6.18	77.35	-0.055	Chloroplast.
*Cluster-21637.2730*	*OjCu/ZnSOD-19*	125	12987.25	5.29	77.92	-0.33	Chloroplast.Cytoplasm.
*Cluster-21637.78187*	*OjCu/ZnSOD-20*	228	23181.32	6.12	90.31	0.109	Chloroplast.
*Cluster-21637.56313*	*OjCu/ZnSOD-21*	160	16604.88	6.57	90.12	0.061	Chloroplast.
*Cluster-21637.76682*	*OjCu/ZnSOD-22*	173	18307.87	5.95	89.02	0.061	Chloroplast.

**Figure 3:**
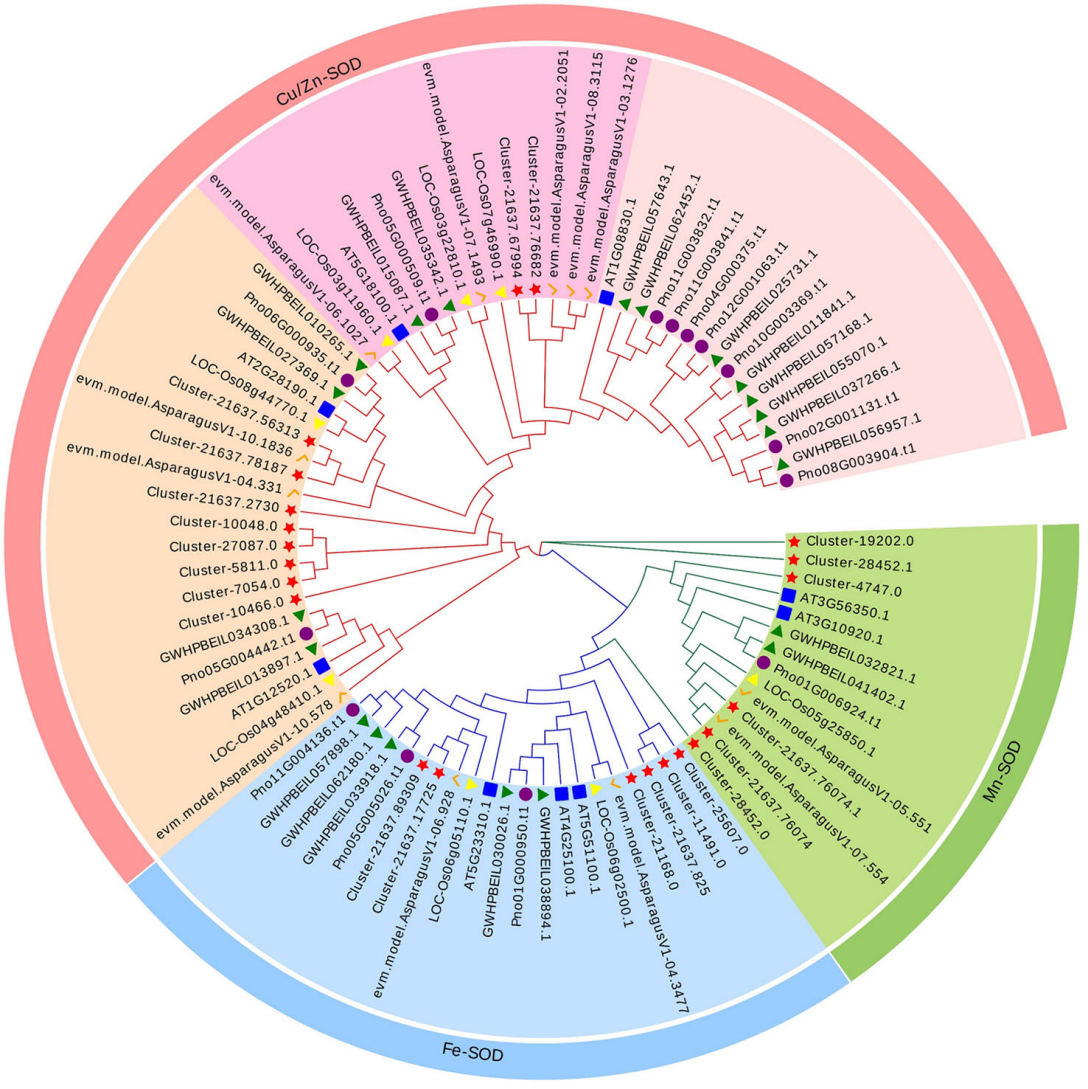
Phylogenetic analysis of 86 SOD proteins from *Ophiopogon japonicus* (red star), *Arabidopsis thaliana* (blue box), *Oryza sativa* (yellow triangles), *Asparagus officinali*s (orange pairs of hooks), *Panax notoginseng* (purple circles) and *Panax ginseng* (green triangles), use iq-tree with 1,000 bootstrap replications.

**Figure 4:**
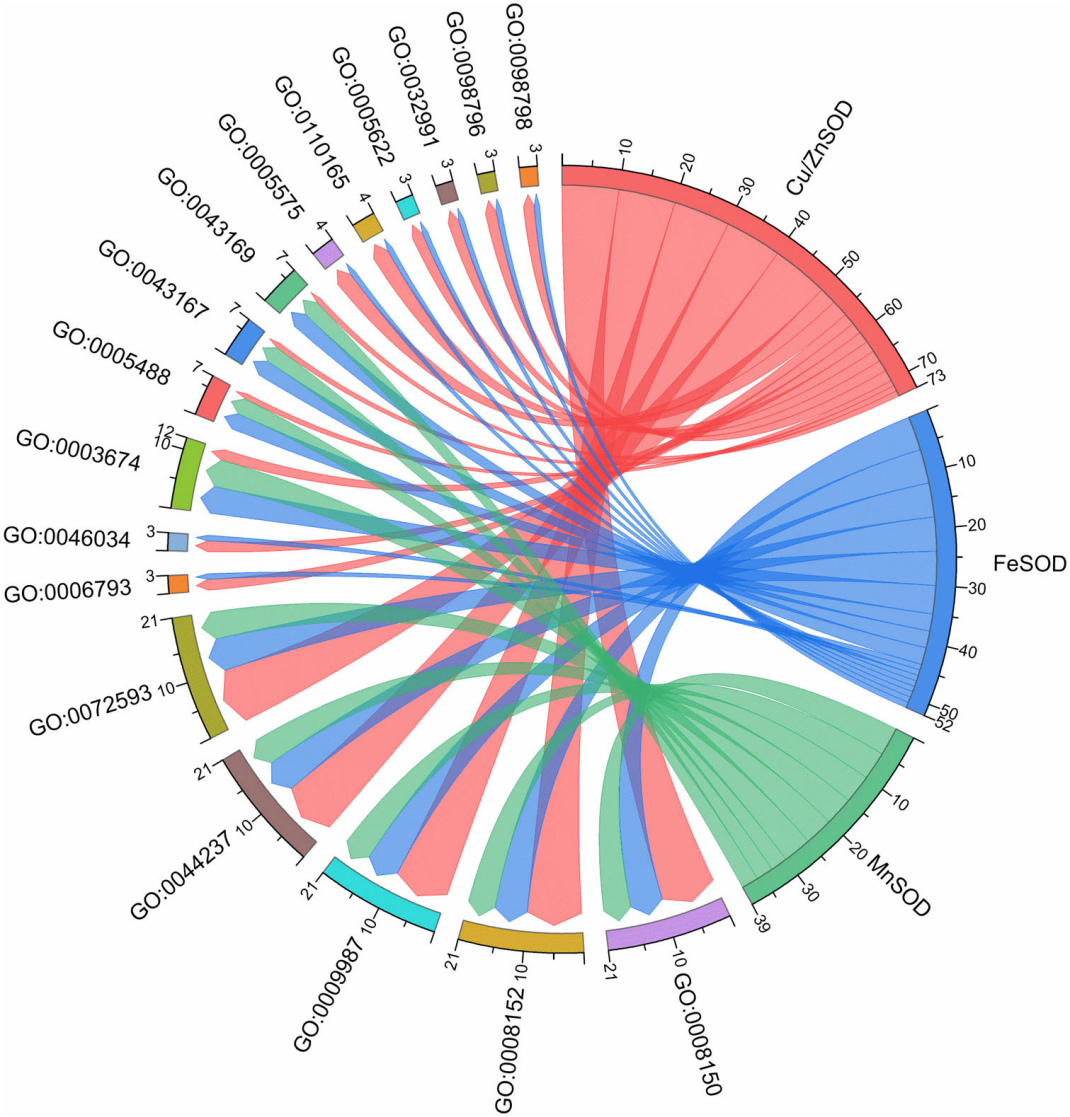
Gene ontology (GO) enrichment of OjSOD proteins.

A phylogenetic tree was constructed in order to explore the evolutionary relationship of SOD from Zhe-Mmaidong in various plant species. The subfamily Cu/ZnSOD exhibited the highest abundance, with a total of 51 members, followed by the FeSOD subfamily, which contained 21 members. The Cu/ZnSOD subfamily could be further divided into three distinct branches. The MnSOD subfamily exhibited the lowest membership count, amounting to a total of 14 (**Figure 3**). Our phylogenetic tree showed that the OjSOD were more closely grouped with SODs from the *A. officinalis* than with proteins from *A. thaliana*. This phenomenon may be explained by the fact that both *O. japonicus* and *A. officinalis* are monocots in the Liliaceae family. To enhance comprehension of the role of *OjSOD* genes, we conducted GO annotation and functionally categorized the *OjSOD* genes of *O. japonicus*. The GO annotation results revealed numerous significantly enriched terms (**Figure 4**), such as biological process (GO:0008150), metabolic process (GO:0008152), cellular process (GO:0009987), cellular metabolic process (GO:0044237), reactive oxygen metabolic process (GO:0072593), molecular function (GO:0003674), binding (GO:0005488), ion binding (GO:0043167), and cation binding (GO:0043169). The majority of *SOD* genes were found to be primarily associated with biological processes (GO:0008150), metabolic processes (GO:0008152), cellular processes (GO:0009987), cellular metabolic processes (GO:0044237), and reactive oxygen metabolism (GO:0072593). The second-highest proportion of *SOD* genes was observed in relation to molecular functions (GO:0003674). In summary, the GO annotation results have provided confirmation of the involvement of *OjSODs* in various stimuli-induced responses, cellular detoxification processes related to oxidants, activities associated with the binding of metal ions, and localization within distinct cellular components.

### 3.3 Analysis of structural domains and conserved motifs of OjSODs

**Figure 5:**
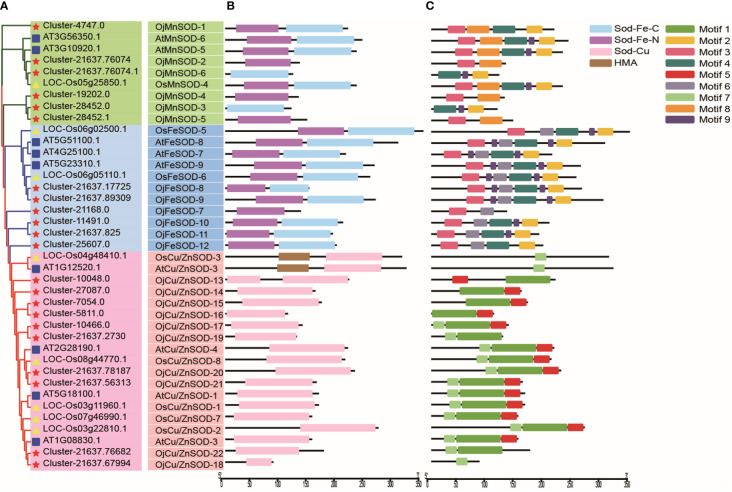
Phylogenetic tree, structural domain, and conserved motifs of *Ophiopogon japonicus*, *Arabidopsis thaliana*, and *Oryza sativa.* **(A)** Phylogenetic relationships of SODs. **(B)** Pfam structural domains of SODs, purple represents SOD_Fe_N structural domain (PF00081), light blue represents SOD_Fe_C structural domain (PF02777), pink represents SOD_Cu structural domain (PF00080), and brown represents HMA structural domain (PF00403). **(C)** Composition of conserved motifs identified in SODs. Different colored boxes indicated different patterns.

Based on the results of structural domain and phylogenetic analysis, the clustering phenomenon observed in FeSODs and MnSODs exhibits a notable degree of similarity, potentially indicating a common ancestral origin. In contrast, Cu/Zn-SODs have evolved separately in eukaryotic organisms, resulting in distinct phylogenetic patterns, as depicted in **Figure 5A**. We identified 19 proteins possessing the Cu/Zn-SOD structural domain (PF00080), 11 proteins containing the Fe/Mn-SOD α-hairpin structural domain (PF00081), and 9 proteins with the Fe/Mn-SOD C-terminal domain (PF02777) (**Figure 5B**). The subfamily classification was closely related to the type of functional domains of the SOD proteins. The classification of SOD proteins into subfamilies is closely associated with their functional domain type. The characteristics of the *OjSOD* family genes were further revealed by performing conserved motif analysis using the MEME server, and 9 conserved motifs were successfully identified. Notably, most closely affiliated individuals within the same subfamily possess a shared motif composition, with a consistent quantity and relative arrangement of these motifs. However, it was observed that the 22 *OjSOD* genes did not exhibit any shared conserved motifs. For example, motif 1, motif 5, and motif 7 exhibited a close association with the Cu/Zn-SOD structural domain (PF00080) and were exclusively observed in the members of Cu/Zn-SOD subfamily; motif 8 demonstrated a relationship with MnSOD and was exclusively present in the MnSOD subfamily; and motif 6 is related to FeSOD and was found only in the FeSOD subfamily. In addition, motif 2, motif 3, motif 4, and motif 9 were specifically observed in OjFeSOD and OjMnSOD proteins, while they were absent in the Cu/Zn-SOD subfamily (**Figure 5C**).

### 3.4 Detection of *OjSOD* genes expression profiles under heavy metal stresses

**Figure 6:**
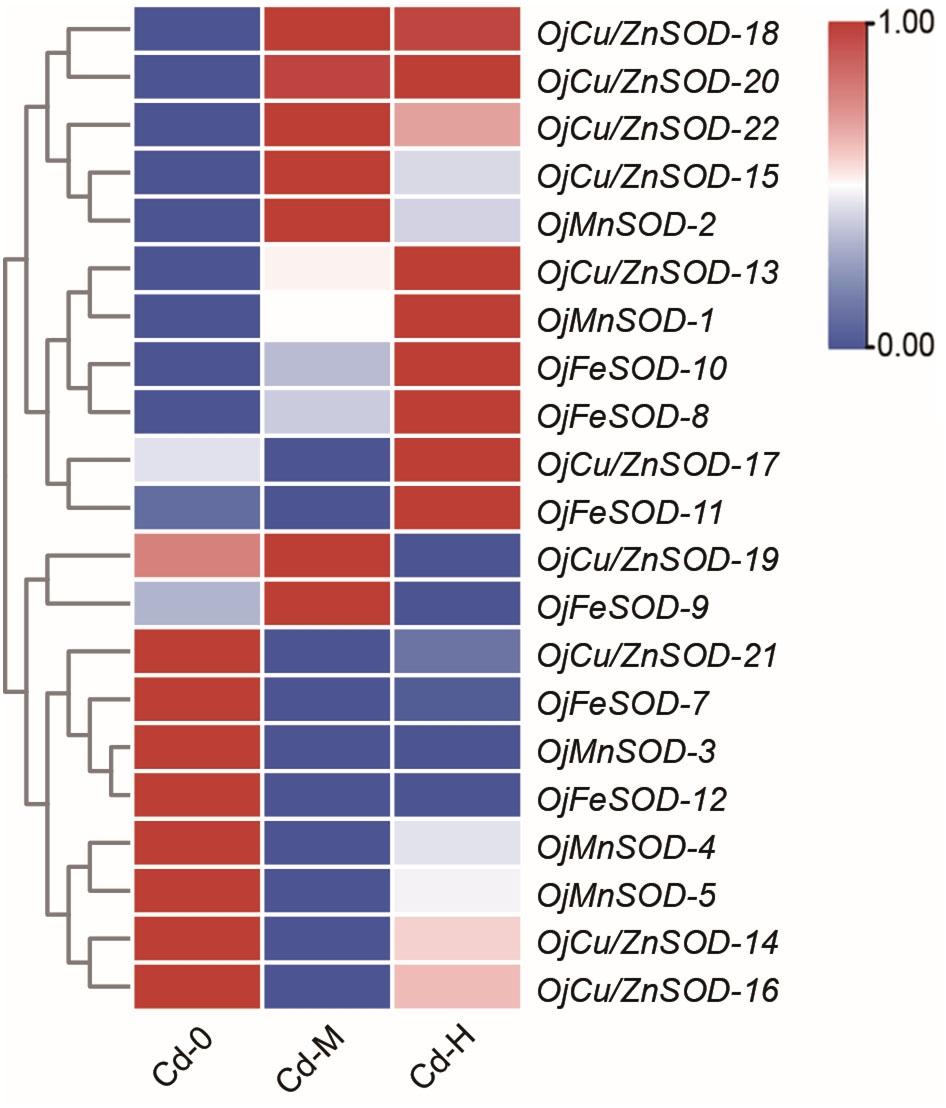
Heat map of *OjSOD* genes expression under Cd exposure. Cd-0, Cd-M, and Cd-H indicated control, 1 mg/L Cd, and 10 mg/L Cd treatments by hydroponics. TBtools was used to generate heatmaps based on (Log_2_(FPKM + 1)) values. Rows normalized the expression of all *OjSOD* genes.

**Figure 7:**
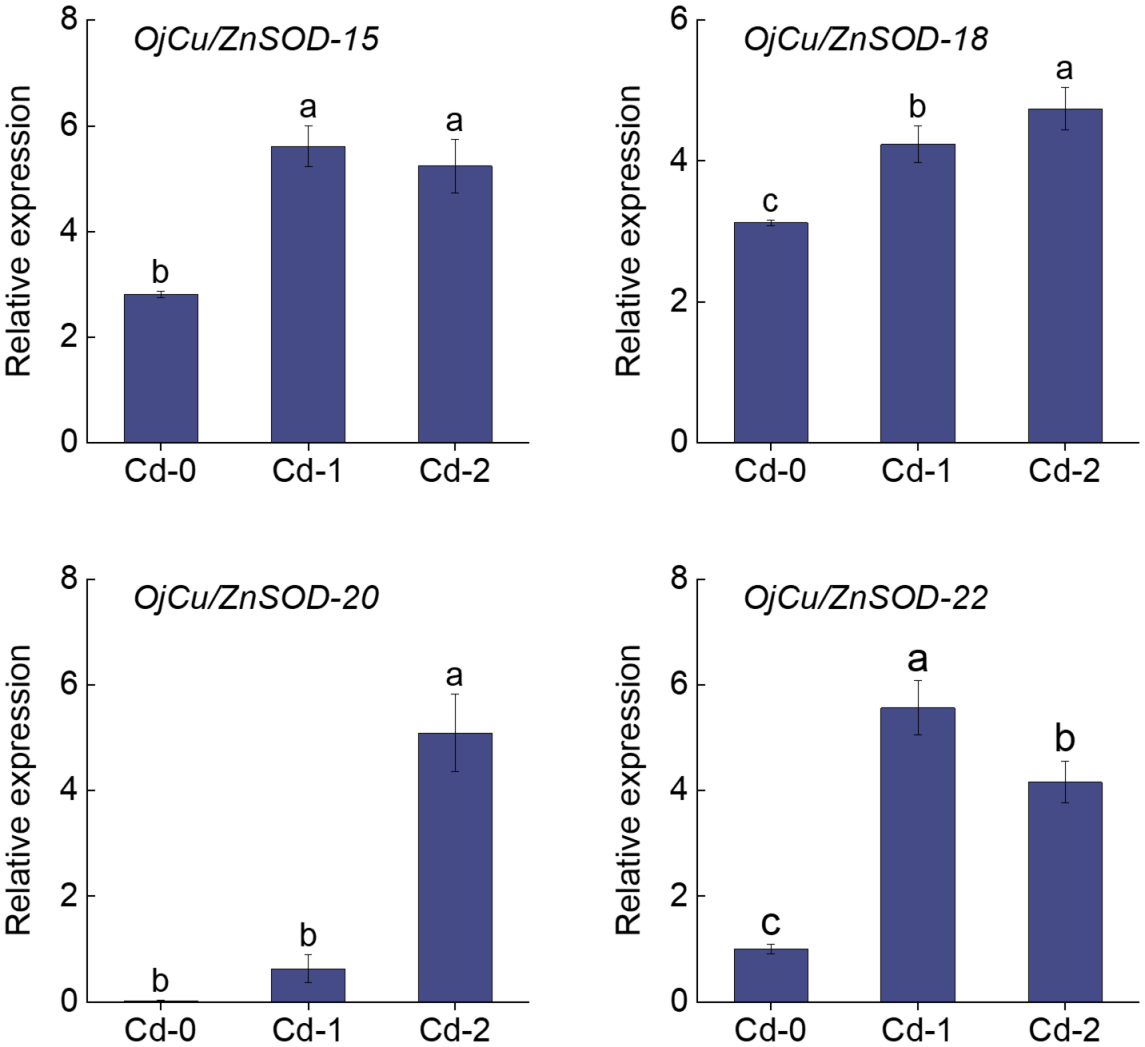
Relative expression levels of *OjCu/ZnSOD-20, OjCu/ZnSOD-22, OjCu/ZnSOD-15*, and *OjCu/ZnSOD-18* under Cd stress by soil cultivation as determined by qRT-PCR. Cd-0, Cd-1, and Cd-2 indicated 0.14 mg/kg Cd, 0.35 mg/kg Cd, and 0.61 mg/kg Cd treatments by soil cultivation. Vertical bars represent standard deviation (SD) from three biological replicates. Different letters indicated significant differences in the LSD test with p <0.05.

The heatmap displayed the findings of the investigation on the variations in the expression of *OjSOD* genes under varying doses of Cd stress. It showed considerable variances in the expression levels of *OjSODs* across different Cd treatments. The cluster analysis results indicate that these genes can be categorized into two groups based on their expression patterns (**Figure 6**). Some genes, including *OjCu/ZnSOD-18*, *OjCu/ZnSOD-20*, *OjCu/ZnSOD-22*, *OjMnSOD-2*, *OjCu/ZnSOD-13*, *OjMnSOD-1*, *OjFeSOD-10*, *OjFeSOD -8*, *OjCu/ZnSOD-17*, *OjFeSOD-11*, *OjCu/ZnSOD-19*, and *OjFeSOD-9*, were up-regulated under Cd stress. On the other hand, the expression of other genes, *OjCu/ZnSOD-21*, *OjFeSOD-7*, *OjMnSOD-3*, *OjFeSOD-12*, *OjMnSOD-4*, *OjMnSOD-5*, *OjCu/ZnSOD-14*, and *OjCu/ZnSOD-16*, decreases under Cd stress. *OjCu/ZnSOD-18*, *OjCu/ZnSOD-20*, *OjCu/ZnSOD-22*, and *OjMnSOD-2* all demonstrated a significant increase in expression under medium and high Cd stress conditions (Cd-M and Cd-H). *OjCu/ZnSOD-13*, *OjMnSOD-1*, *OjFeSOD-10*, *OjFeSOD-8*, *OjCu/ZnSOD-17*, and *OjFeSOD-11* only demonstrated a significant increase in expression levels under severe Cd stress (Cd-H). Furthermore, *OjCu/ZnSOD-19* and *OjFeSOD-9* exhibited enhanced expression on their own in response to moderate Cd stress (Cd-M). To verify the accuracy of the transcriptome data, a total of 11 genes were randomly selected for qRT-PCR verification. The gene expression patterns detected from qRT-PCR were consistent with the transcriptome data, suggesting a high confidence level in the data obtained from the transcriptome sequencing (**Supplementary Figure 1**). Additionally, we conducted gene expression analysis of *OjCu/ZnSOD-20*, *OjCu/ZnSOD-22*, *OjCu/ZnSOD-15*, and *OjCu/ZnSOD-18* genes in response to various soil culture treatments with Cd stresses. The results indicated that Cd stress significantly elevated the expression levels of these four genes under the soil cultivation conditions as well (**Figure 7**). Based on the results of physiological detection, it was preliminarily hypothesized that *OjCu/ZnSOD-20, OjCu/ZnSOD-22, OjCu/ZnSOD-15*, and *OjCu/ZnSOD-18* perform a vital role in encoding the Cu/ZnSODa and Cu/ZnSODb isozymes under the Cd-stress treatment.

**Figure 8:**
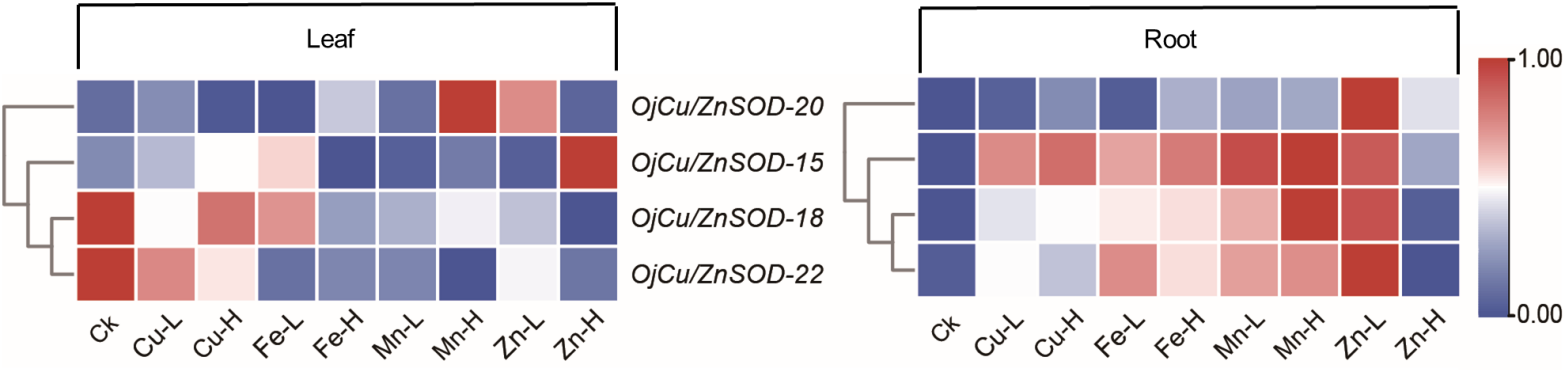
Expression of *OjSOD* genes under heavy metal stress in different tissues of Zhe-Maidong by soil cultivation. TBtools was used to generate heatmaps based on (log_2_(qPCR+1)) values. Rows normalized the expression of all *OjSOD* genes.

The expression patterns of the four *OjSOD* genes *Cu/ZnSOD-20, Cu/ZnSOD-22, Cu/ZnSOD-15*, and *Cu/ZnSOD-18*, which exhibited significant up-regulation in response to Cd stress, were investigated further in the presence of additional heavy metal stresses (Cu^2+^, Fe^2+^, Zn^2+^, Mn^2+^). As shown in **Figure 8**, the expression of *OjSOD* candidate genes in leaves and roots showed obvious organ differentiation under different heavy metal stress treatments, with most of the genes down-regulated in leaves. They upregulated in roots (*Cu/ZnSOD-15, Cu/ZnSOD-18*, and *Cu/ZnSOD-22*). The expression of *OjSOD* candidate genes in leaves and roots exhibited distinct organ variations under various heavy metal stresses. The majority of genes exhibited a decrease in expression levels in leaves and an increase in expression levels in roots. In the roots, these four candidate genes were consistently expressed at higher levels in the roots under various metal stressors, including Cu-L (10 mg/Kg), Cu-H (100 mg/Kg), Fe-L (2 mg/Kg), Fe-H (20 mg/Kg), Mn-L (10 mg/Kg), Mn-H (100 mg/Kg), Zn-L (25 mg/Kg), and Zn-H (250 mg/Kg). Among the four candidate genes, *Cu/ZnSOD-15, Cu/ZnSOD-18*, and *Cu/ZnSOD-22* exhibited a more pronounced up-regulation effect, indicating that they might have a crucial function in responding to heavy metal stresses. The gene expression of *Cu/ZnSOD-18* and *Cu/ZnSOD-22* exhibited a decrease in response to high-content zinc stress, which could potentially be attributed to the zinc ion concentration surpassing the tolerance range of the plant. These results suggest that *Cu/ZnSOD-20, Cu/ZnSOD-22, Cu/ZnSOD-15*, and *Cu/ZnSOD-18* might benefit the ability of *O. japonicus* to cope with heavy metal-induced stress.

### 3.5 Prediction of the 3D structure of OjSODs with their key residues

**Figure 9:**
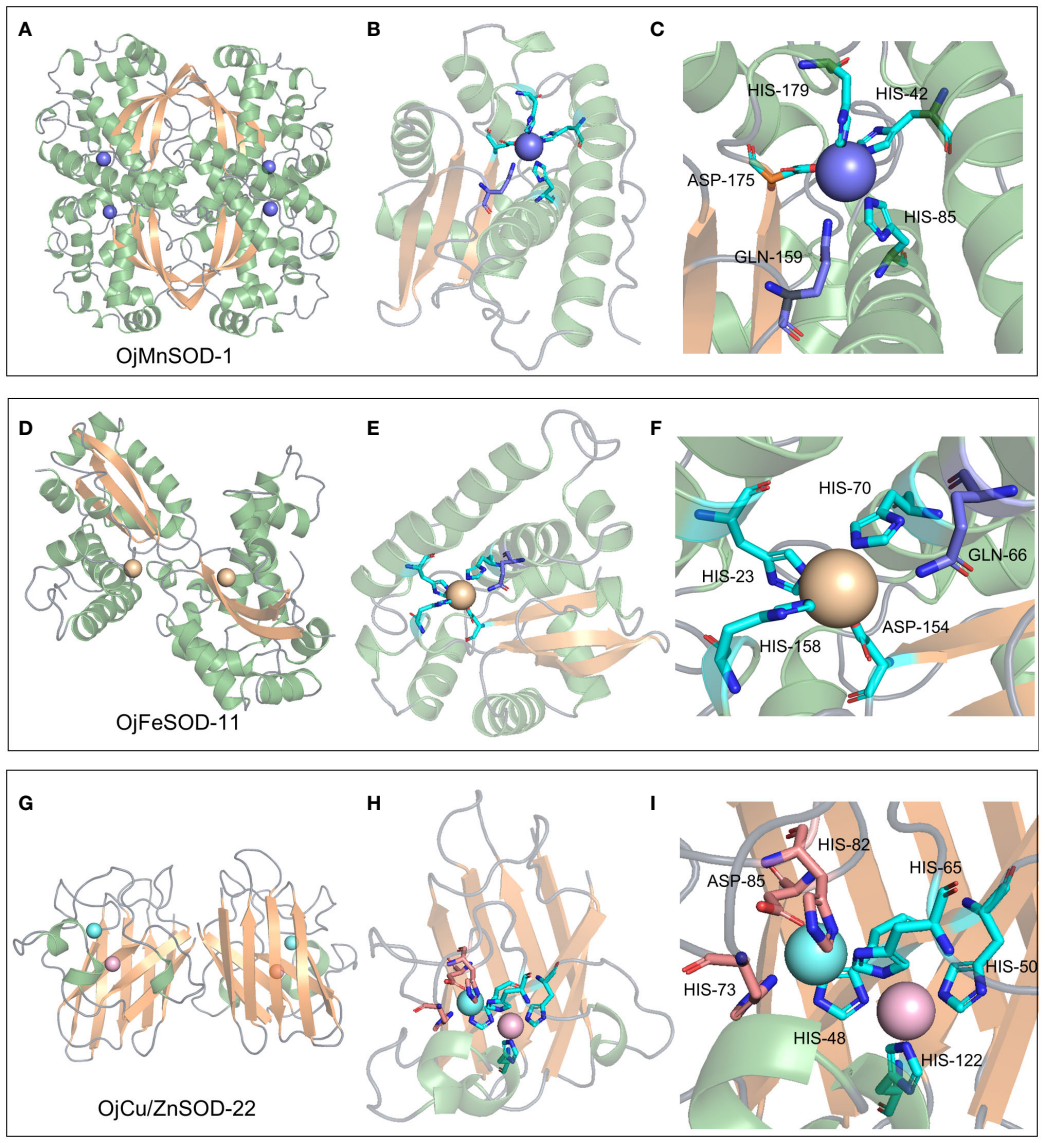
3D structure, metal ion binding site, and metal ion binding residues of OjMnSOD-1 **(A-C)**, OjFeSOD-11 **(D-F)**, and OjCu/ZnSOD-22 **(G-I)**.

**Figure 10:**
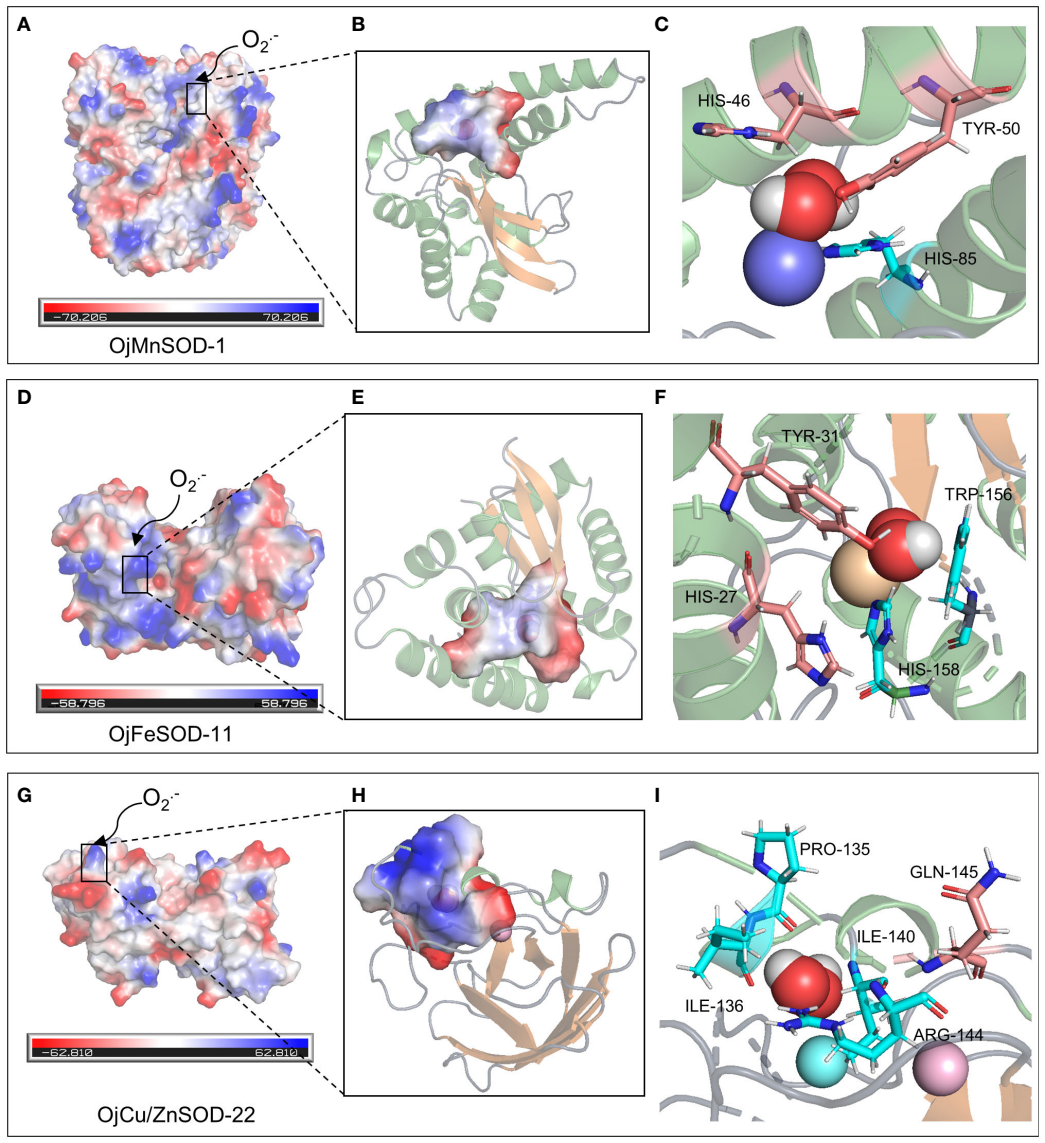
Molecular docking of superoxide anion with three OjSOD proteins. Active site, O_2_^•−^ binding pocket, O_2_^•−^ binding residues, and the orientation into the substrate of OjMnSOD-1 **(A-C)**, OjFeSOD-11 **(D-F)**, and OjCu/ZnSOD-22 **(G-I)**.

A 3D model of the SOD proteins was successfully obtained by comprehensively analyzing the comparison coverage, percentage of identity, and confidence level of the sequences to be tested using the templates in the database on the SWISS-MODEL website (**Supplementary Figure 2**). OjMnSOD-1 and OjMnSOD-6 existed as homotetramers, whereas OjMnSOD-2, OjMnSOD-4, OjMnSOD-5, and OjFeSOD-7 were in the form of monomers. The rest of the SOD proteins were homodimers. To validate the predicted models, we evaluated their stereochemical quality by utilizing Ramachandran plots. The results showed that in all models, the majority of residues were located in the most favorable and permissive regions, accounting for proportions ranging from 90.48% to 98.80% (**Supplementary Table S3**, **Supplementary Figure 3**). The forbidden regions exhibited a scarcity or absence of residues, suggesting the high geometry of the models. To specify the crucial residue substrates involved in the catalytic substrates of SOD proteins, we performed molecular docking experiments of O_2_^•−^ using one representative sample from each of the three types of SOD isoforms (**Figures 9**, **10**). Initially, we assessed the docking results’ stability and the binding strength between the ligand and the receptor by utilizing the -CDocker Energy and -CDocker Interaction Energy values obtained during molecular docking (**Supplementary Table S4**). The higher -CDocker Energy gives the receptor and ligand better binding affinity. Similarly, as the value of -CDocker Interaction Energy increases, the energy needed for the interaction force between the ligand and the receptor decreases, resulting in a stronger binding state between the ligand and the receptor. Our experimental results indicated that OjCu/ZnSOD-22 exhibits a superior score, suggesting a robust binding activity between OjCu/ZnSOD-22 and superoxide anion.

The biological function and interactions of a protein are determined by its structure, with the binding sites typically being conserved in both sequence and structure (Lin et al., 2016). Thus, we performed a comparative analysis of various sequences with previously documented humans and cows and identified that His48, His50, His65, and His122 of the OjCu/ZnSOD-22 protein from *O. japonicus* exhibit a high degree of conservation and serve as crucial amino acid sites for binding Cu^2+^, suggesting that these four amino acid residues play an essential role in binding Cu^2+^. Furthermore, by examining the conservation of the Zn^2+^ binding site, we have determined the crucial residues His65, Asp85, His73, and His82 for the function of Zn ion binding. (**Figure 9I**). Among them, a key functional role in the substrate binding process was played by GLN-145, while the binding site for the superoxide anion was constituted by Pro135, Ile136, Arg144, and Ile140 (**Figure 10I**). Furthermore, we suggest that Gln145 exerts a vital functional role in the process of substrate binding, whereas Pro135, Ile136, Arg144, and Ile140 form the specific location wherein the superoxide anion binds.

Similarly, 3D structural models of MnSOD and FeSOD were constructed, and it was observed that they have similar folded morphologies to MnSOD and FeSOD structures determined in other species. Based on multiple sequence alignments analysis, we hypothesize that the Mn metal binding site in OjMnSOD-1 is coordinated by specific amino acid residues: His42, His85, His179, and Asp175, along with an oxygen-containing molecule such as water or hydroxide (**Figure 9C**). The substrate enters the active site by processes of assisted diffusion involving His46 and Tyr50. Once within, the substrate binds to His85 (**Figure 10C**). In the case of OjFeSOD-11, the Fe metal is bound to the amino acid residues His23, His70, His158, Asp154, and a molecule containing oxygen (**Figure 9F**) (Zhao et al., 2021). The substrate diffuses and enters the active site through the synergistic interaction of Tyr31 and His27, while His158 and Trp156 served as the binding sites for the superoxide anion (**Figure 10F**).

## 4 Discussion

Plants possess antioxidant enzyme systems that efficiently protect cells from reactive oxygen species (ROS) damage and enhance their ability to resist adverse stresses (Semida et al., 2018). The adverse impacts of Cd stress on plants are evident through the induction of growth inhibition and disruption of photosynthesis and respiration activities (Tran and Popova, 2013). Our investigation showed that Cd stress led to an increase in O_2_^•−^, H_2_O_2_, and MDA content in both roots and leaves of Zhe-Maidong, resulting in oxidative damage (**Figure 1**). The SOD activity in the roots of Zhe-Maidong exhibited a significant increase under Cd stress, contrasting with the response of other antioxidant enzymes, such as APX and CAT. Variations in the responses of SOD isoenzymes to adverse stresses are substantial across different species or organs. To illustrate, when *P. sativum* L. was subjected to a Cd treatment at a concentration of 50 μM, the activity of Cu/ZnSOD in the roots was suppressed, MnSOD exhibited increased activity, and the FeSOD activity remained constant (Rodríguez Serrano et al., 2006). However, the SOD isozyme activities were significantly decreased in *Pisum sativum* L. leaves under the same treatment conditions. Under conditions of water stress in *Lotus japonicus*, there was a slight rise in the activities of MnSOD and Cu/ZnSOD (I and II) in the roots, whereas the activity of FeSOD decreased. Nevertheless, the activity of the three SOD isoenzymes in leaves remained constant (Signorelli et al., 2013). Our study revealed that the SOD isoenzymes of Zhe-Maidong also exhibited distinct organ-specific responses to Cd stress (**Figure 2**). Based on the results of SOD isoenzyme physiological measurement and Native-PAGE detection, Cu/ZnSOD contributed 58.56% to 62.38% of the total SOD enzyme activity in the roots under Cd stress. Furthermore, it was observed that the enzyme activities Cu/ZnSODa and Cu/ZnSODb exhibited a substantial increase in response to various Cd stress regimens. Notably, the amount of the increase in Cu/ZnSODa and Cu/ZnSODb enzyme activities was much higher than that of Fe/MnSOD. The tissue-specific expression of SODs in response to Cd stresses in Zhe-Maidong or other plants is a result of complex regulatory mechanisms that are influenced by various factors. Here, we supposed that tissue-specific expression of SOD isozymes helps to maintain the ROS balance, ensuring proper cellular function in the leaf or root with distinct metabolic demands (Che et al., 2020). On the other hand, it possibly resulted from regulation by metabolites or other regulatory factors (Chu et al., 2005; Du et al., 2023; Zheng et al., 2023). Overall, these results validate that Cu/ZnSODa and Cu/ZnSODb were the primary components responsible for the rise in total SOD enzyme activity when subjected to Cd stress and played a crucial role in the scavenging of Cd stress-induced ROS damage.

Twenty-two *OjSOD* genes were identified based on the transcriptome data to determine the pivotal genes responsible for regulating alterations in SOD activity during Cd stress. In accordance with their domains and patterns, they were classified into three subfamilies: Cu/ZnSOD, FeSOD, and MnSOD. This categorization aligns with the classifications in *Nicotiana tabacum* (Huo et al., 2022), *Salvia miltiorrhiza* (Han et al., 2020), soybeans (Aleem et al., 2022), *Daucus carota* (Zameer et al., 2022), potato (Rudić et al., 2022), and wheat (Tounsi et al., 2023). The gene family of SOD in Zhe-Maidong consists of 10 *OjCu/ZnSOD*, 6 *OjMnSOD*, and 6 *OjFeSOD* genes. It is noteworthy to mention that the Cu/Zn-SOD subfamily has a greater number of members compared to the Fe-SOD or Mn-SOD subfamily, implying that the Cu/Zn-SOD subfamily may be indispensable for plant growth, development, and stress response. The expression levels of *OjCu/ZnSOD-20*, *OjCu/ZnSOD-22*, *OjCu/ZnSOD-15*, and *OjCu/ZnSOD-18* was significantly upregulated under Cd stress, which was consistent with increased Cu/ZnSODa and Cu/ZnSODb activities despite of soil or hydroponic culture. Thus, we proposed that the significant up-regulation of *OjCu/ZnSOD-20*, *OjCu/ZnSOD-22*, *OjCu/ZnSOD-15*, and *OjCu/ZnSOD-18* expression levels was the primally contributing to the enhanced activities of Cu/ZnSODa and Cu/ZnSODb. Subsequently, we performed the expression assays of *OjCu/ZnSOD-20*, *OjCu/ZnSOD-22*, *OjCu/ZnSOD-15*, and *OjCu/ZnSOD-18* genes in response to heavy metal stresses, including Cu^2+^, Fe^2+^, Zn^2+^, and Mn^2+^. The findings revealed that the *OjSOD* genes in roots were responsive to various heavy metal stresses, with *Cu/ZnSOD-15, Cu/ZnSOD-18, and Cu/ZnSOD-22* exhibiting significantly increased expression levels (**Figure 8**). This aligns with prior research findings in rice, which reported that the expression of *OsCu/ZnSOD-3* was markedly increased in response to Cd stress, serving as a defense mechanism against the toxic effects of Cd (Yang et al., 2020). Similarly, Liu et al. found a notable increase in the expression of *Cu/ZnSOD* under Cd treatment in rice (Liu et al., 2023). In chickpea seedlings, it was also observed that Cd stress induced upregulated expression of *Cu/Zn-SOD* in roots (Sakouhi et al., 2023). In addition, phylogenetic analyses revealed a close homologous association between the *Cu/ZnSOD* genes of Zhe-Maidong and *Cu/ZnSOD* of rice. Therefore, it may be inferred that the genes *Cu/ZnSOD-15*, *Cu/ZnSOD-18*, and *Cu/ZnSOD-22*, similar to the *OsCu/ZnSOD-3* gene in rice, possess an important function in resisting Cd and other metal ion stresses.

The structure of a protein determines its biological role and interactions. Comparison with previously identified homologous protein sequences and 3D structures demonstrated that Cu/ZnSOD was a very conserved protein, exhibiting conservation in its primary structure, crucial residue locations, domain organization, and tertiary structure (Perry et al., 2010). OjCu/ZnSOD-22 was composed of a homotetramer, similar to the previous report that Cu/ZnSOD consists of a β-barrel of eight antiparallel chains grouped in a Greek bonding pattern, which helps stabilize the structure of the entire protein (Tainer et al., 1982; Rudić et al., 2022). The primary role of SOD was to catalyze the disproportionation of H_2_O_2_ and O_2_^•−^ by superoxide anion radicals (Sawyer and Valentine, 1981). For Cu/ZnSOD, the enzymatic reaction occurs at the copper ion in two steps: first, Cu^+^ reduces O_2_^•−^ to H_2_O_2_ and Cu^2+^, and then the Cu^2+^ oxidizes another O_2_^•−^ to form O_2_ and returns to the Cu^+^ (Furukawa, 2021). According to the homologous comparison, we found the key residue sites for the catalytic reaction of OjCu/ZnSOD-22 from *O. japonicus*. When the copper ion is in the +1 oxidation state, it could form a triangular structure by binding Nδ1 of His48, Nϵ2 of His50, and His122. Up oxidation of the copper ion to the +2 state, it would combine with Nϵ2 of His65 and a water molecule, forming a distorted square pyramidal geometry. Nδ1 of His65 binds to the zinc ion and forms a tetrahedral coordination with the carboxylate of Asp85 and Nδ1 of His73 and His82 (**Figure 9I**). His65 could function as a bridge, binding Cu^2+^ and Zn^2+^ (Furukawa, 2021). Copper and zinc binding sites were required, which were conserved in all Cu/ZnSODs, and copper and zinc ions had critical functions in stabilizing the quaternary structure to increase the probability of pathogenic mutants (Lynch and Colón, 2006; Ni et al., 2007; Anju et al., 2013; He et al., 2014). The active core of Cu/ZnSOD contains five conserved histidines that have been identified as crucial for sustaining the enzymatic activity of SOD. When the five His active sites of Cg-CuZn-SOD undergo mutations, such as replacing 49His with Gln, 66His with Leu, 74His with Asn, 83His with Asn, and 123His with Leu, it leads to a loss of SOD enzyme activity, providing further evidence of the critical role played by these conserved residues in maintaining SOD enzyme activity (Ruan et al., 2022). Through multiple sequence alignments, we found that at the corresponding positions of OjCu/ZnSOD-22 are residues His48, His65, His73, His82, and His122, which we hypothesize also play a key role in maintaining enzyme activity. The multiple sequence alignment reveals that the conserved active sites in OjCu/ZnSOD-22 were His48, His65, His73, His82, and His122. It was hypothesized that they have a crucial function in preserving the activity of the SOD enzyme in Zhe-Maidong.

MnSOD and FeSOD originated from common ancestral genes, and their products are often referred to as Fe/MnSODs (Wang et al., 2017). Both of these two isozymes possessed the same conserved structural domains, namely the α-folding structural domain (PF00081) and the C-terminal structural domain (PF02777), which were part of Fe/MnSOD (**Figure 5**). Previous research showed that certain structural characteristics could distinguish MnSODs from FeSOD isoenzymes in Zhe-Maidong (Bonetta Valentino, 2022; Rudić et al., 2022). As shown in **Supplementary Figure 4**, FeSOD sequences represented by OjFeSOD-11 possess the Q residue at position 66, which actively engages in hydrogen bonding with water. However, in the OjMnSOD represented by OjMnSOD-1 protein, the Q residue was situated at the 159th amino acid position. The slight variation between the two Q amino acid sites affected the redox regulation of SOD activities and determined whether the enzyme binds Mn or Fe. Homology modeling showed that *OjMnSOD-1* was composed of a homotetramer, and each subunit contains an active site with manganese as a cofactor (**Figure 9C**) (Rudić et al., 2022). The results of comparing the sequence with humans displayed that the Mn^2+^ was coordinated by residues His42, His85, His179, Asp175, and an oxygen-containing molecule such as water or hydroxide (Abreu and Cabelli, 2010; Azadmanesh et al., 2017). According to Azadmanesh and Borgstahl, we supposed that the substrate would diffuse through His46 and Tyr50 to the active site, where it forms coordination bonds with Mn cofactors (**Figure 10C**) (Azadmanesh and Borgstahl, 2018). Mn could embedded in the active site cavity, which contains a hydrogen-bonding network that facilitates proton transfer in the reduction of O_2_^•−^ to H_2_O_2_ with two key residues, tyrosine and glutamine. In human MnSOD, these two amino acids are Y34 and Q143, respectively (Abreu and Cabelli, 2010; Bonetta Valentino, 2022). According to the sequence comparison, the equivalent residues of OjMnSOD-1 in Zhe-Maidong were Tyr50 and Gln159. These results provide evidence that Tyr50 and Gln159 residues in MnSOD were crucial for preserving enzyme function. As determined by comparing the sequence to *A. thaliana* and *Vigna angularis*, the Fe was coordinated by residues His36, His84, His171, Asp167, and an oxygen-containing molecule. The substrate could diffuse through Tyr31 and His27 and enter the active site, coordinating with the iron cofactor (**Figure 10F**). Although the active sites of FeSOD and MnSOD were structurally similar, the introduction of Mn ions into the active site of iron SOD leads to a decrease in dismutase activity and vice versa (Abreu and Cabelli, 2010). In human MnSOD, the His30 mutation restricts substrate entry into the substrate funnel and interferes with superoxide binding, resulting in a reduced catalytic rate and increased product inhibition (Hearn et al., 2003). The Q143 mutant alters the metal specificity and active site redox potential, decreasing its catalytic activity (Lévêque et al., 2000). By multiple sequence alignment, we found that His46 and Q159 occupy the corresponding positions in OjMnSOD-1 (**Supplementary Figure 4**). Therefore, it was reasonable to speculate that these two residues might play a substantial and crucial role in maintaining the catalytic rate. Further, targeted mutagenesis investigations are required to thoroughly explore their important roles in enzyme activity.

## 5 Conclusions

Cd stress resulted in a notable elevation in the SOD activity in the roots of Zhe-Maidong. The most prominent SOD isozymes responding to Cd stress were Cu/ZnSODa and Cu/ZnSODb. Via the investigation of conserved motif and phylogenetic tree, a total of 22 *OjSOD* genes were identified and classified into three subgroups: 10 *OjCu/ZnSODs*, 6 *OjMnSODs*, and 6 *OjFeSODs*. Of all the 22 *SOD* genes, *Cu/ZnSOD-15*, *Cu/ZnSOD-18*, *Cu/ZnSOD-20*, and *Cu/ZnSOD-22* were the main genes that control the increase in SOD activity under Cd stress. Key residues of OjSOD proteins were found through multiple sequence alignment and molecular docking. The conserved active sites in OjCu/ZnSOD-22 were His48, His65, His73, His82, and His122. In addition, Gln145 exerts a vital functional role in the process of substrate binding, whereas Pro135, Ile136, Arg144, and Ile140 form the specific location wherein the superoxide anion binds. Additional focused mutagenesis studies are necessary to comprehensively investigate their significant contributions to enzyme activity.

## Data availability statement

The original contributions presented in the study are included in the article/**Supplementary Material**, further inquiries can be directed to the corresponding author/s.

## Author contributions

RH: Methodology, Writing – original draft, Writing – review & editing. ZW: Formal analysis, Methodology, Writing – original draft. QZhu: Formal analysis, Methodology, Writing – original draft. JW: Formal analysis, Methodology, Writing – original draft. YZ: Supervision, Writing – review & editing. YL: Supervision, Writing – original draft. HL: Methodology, Writing – original draft. QZha: Methodology, Supervision, Writing – original draft, Writing – review & editing. JH: Supervision, Writing – review & editing.
