# Supplementary material for: Identification and characterization of the critical genes encoding Cd-induced enhancement of SOD isozymes activities in Zhe-Maidong (Ophiopogon japonicus)
Source: Front Plant Sci. 2024 Mar 28;15:1355849. doi: 10.3389/fpls.2024.1355849 (PMC11007131; doi:10.3389/fpls.2024.1355849)
Supplement: Supplementary file 1 [file DataSheet_1.pdf]

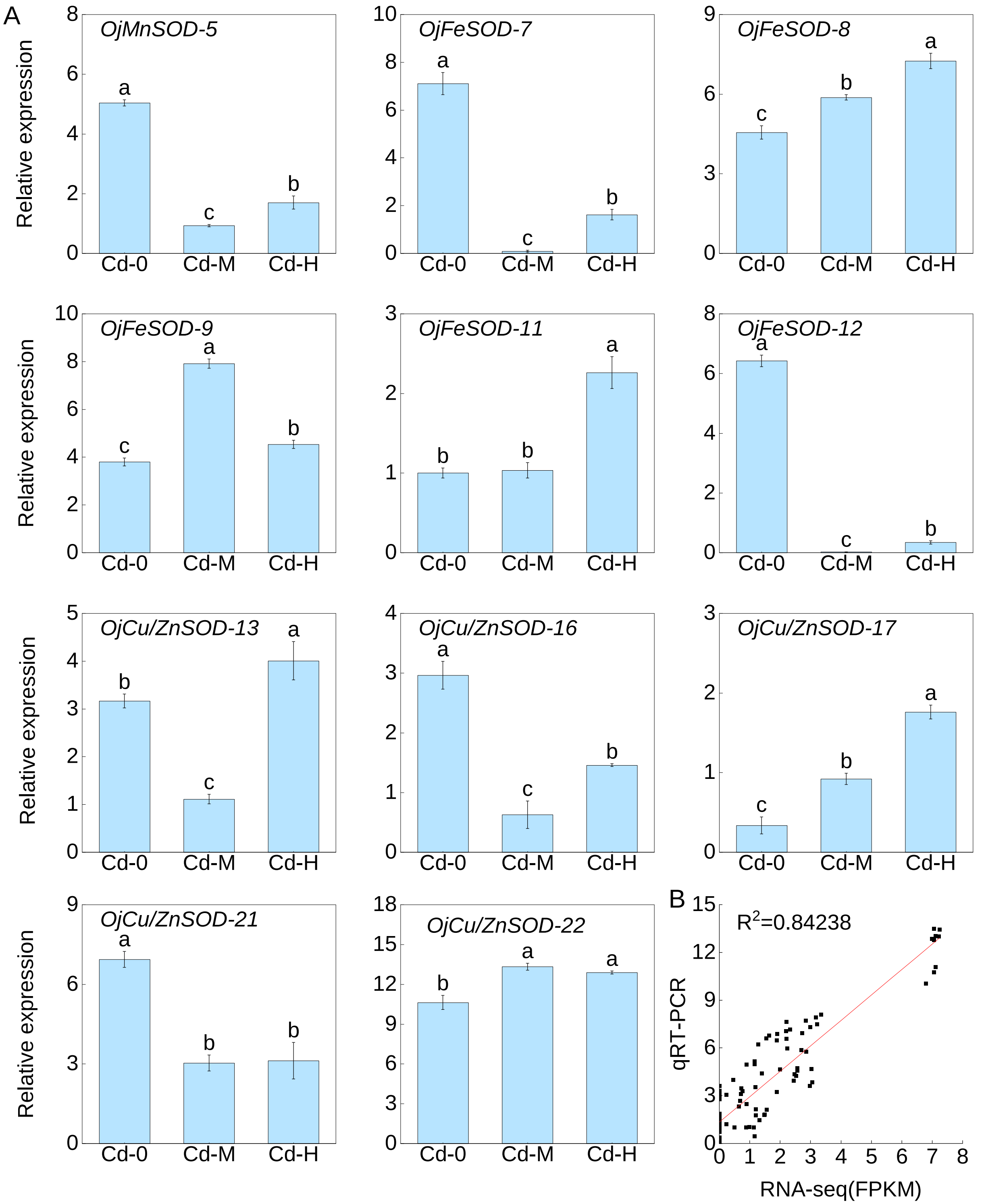

A
OjMnSOD-5
OjFeSOD-7
OjFeSOD-8
OjFeSOD-9
OjFeSOD-11
OjFeSOD-12
OjCu/ZnSOD-13
OjCu/ZnSOD-16
OjCu/ZnSOD-17
OjCu/ZnSOD-21
OjCu/ZnSOD-22
Relative expression
Cd-0
Cd-M
Cd-H
B
R^2=0.84238
qRT-PCR
RNA-seq(FPKM)

Supplementary Figure 1 The correlation coefficient of the SODs expression pattern obtained by RNA-Seq and qRT-PCR. (A). Relative expression levels of eleven SODs under Cd stress as determined by qRT-PCR. The treatments were Cd-0, Cd-M, and Cd-H.(B). Compare the log_2(value+1) of gene expression data from RNA-seq data and qRT-PCR validation data. R^2 represented the correlation coefficient between RNA-Seq data and qRT-PCR validation data.

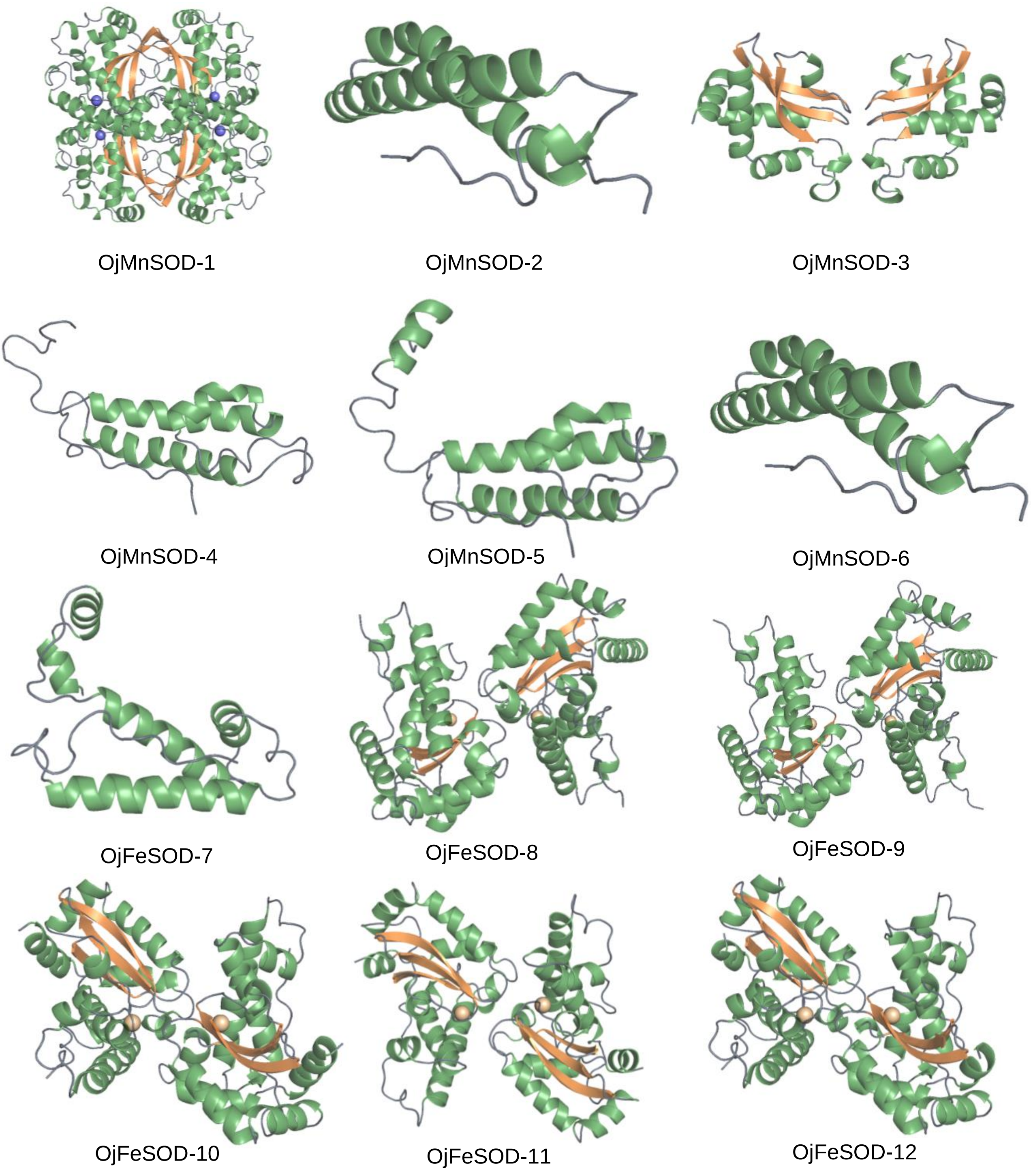
OjMnSOD-1
OjMnSOD-2
OjMnSOD-3
OjMnSOD-4
OjMnSOD-5
OjMnSOD-6
OjFeSOD-7
OjFeSOD-8
OjFeSOD-9
OjFeSOD-10
OjFeSOD-11
OjFeSOD-12

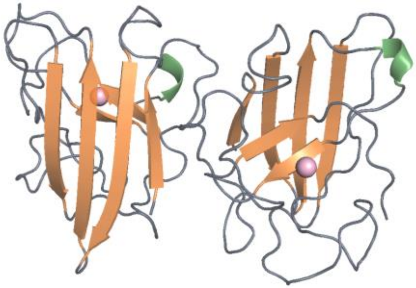

OjCu/ZnSOD-13

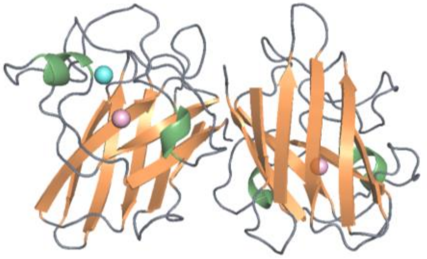

OjCu/ZnSOD-14

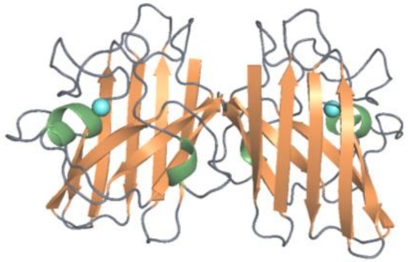

OjCu/ZnSOD-15

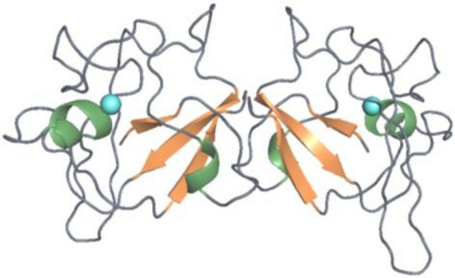

OjCu/ZnSOD-16

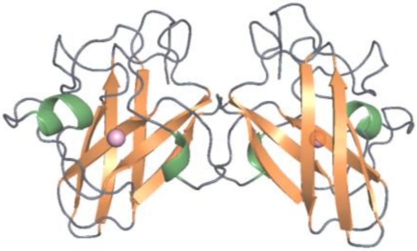

OjCu/ZnSOD-17

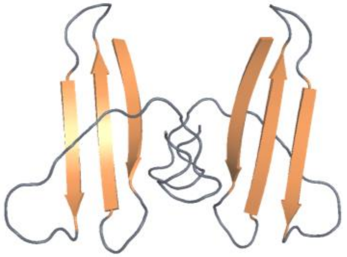

OjCu/ZnSOD-18

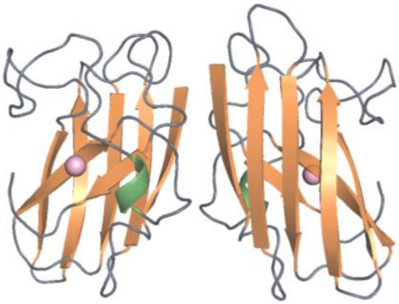

OjCu/ZnSOD-19

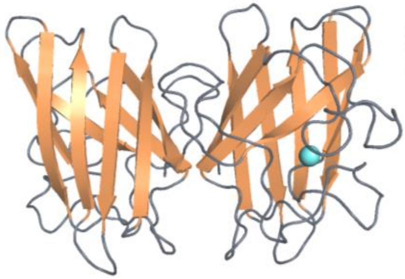

OjCu/ZnSOD-20

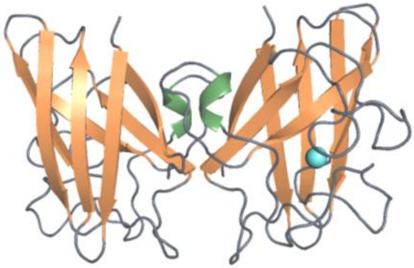

OjCu/ZnSOD-21

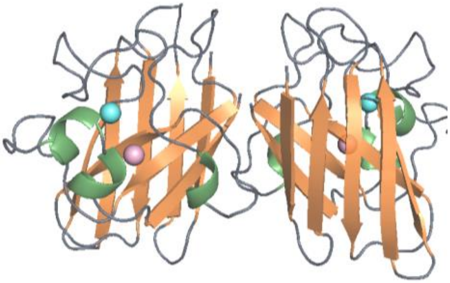

OjCu/ZnSOD-22

Supplementary Figure 2 3D structure of OjSOD proteins.

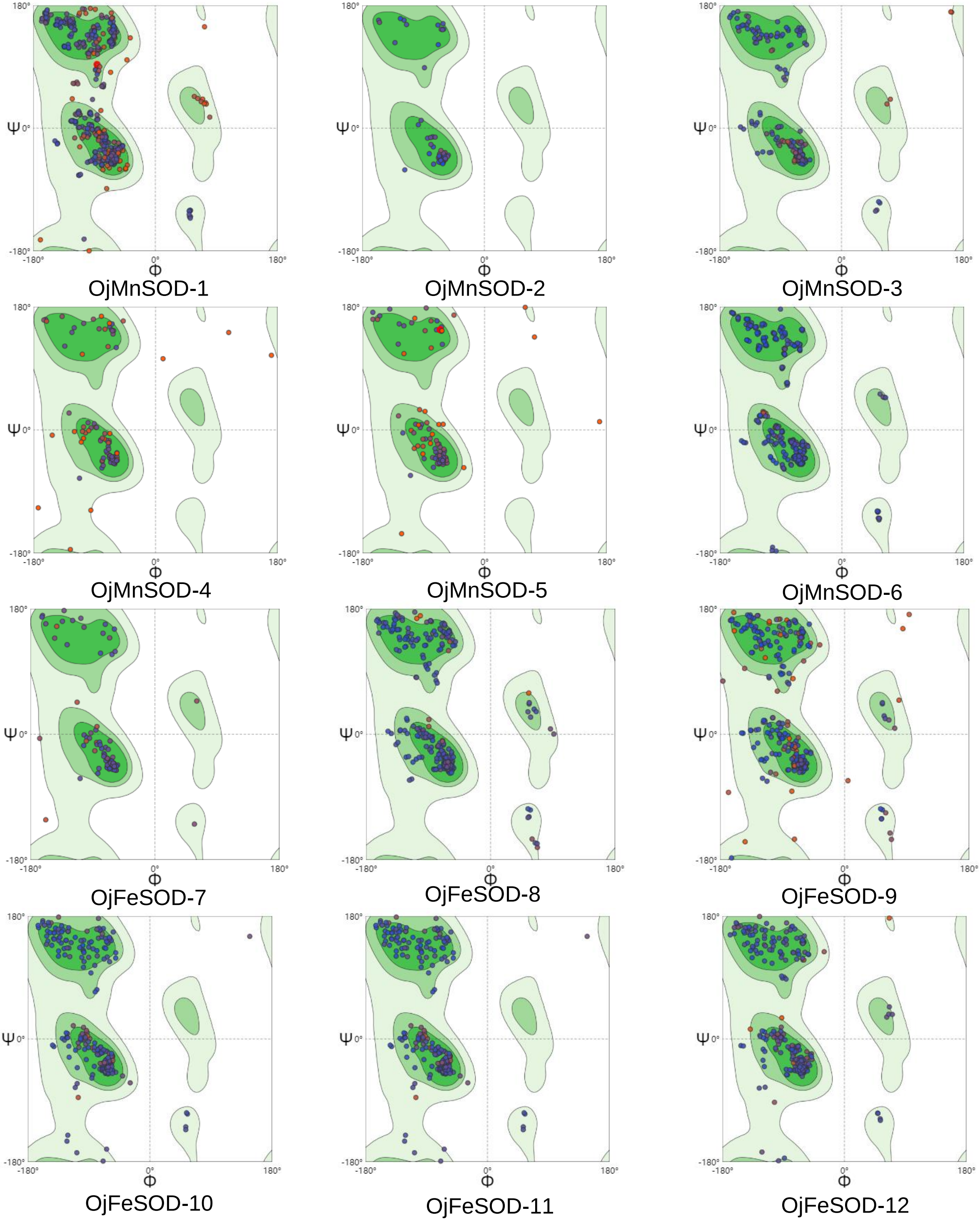

OjMnSOD-1
OjMnSOD-2
OjMnSOD-3
OjMnSOD-4
OjMnSOD-5
OjMnSOD-6
OjFeSOD-7
OjFeSOD-8
OjFeSOD-9
OjFeSOD-10
OjFeSOD-11
OjFeSOD-12
Φ
Ψ
180°
0°
-180°

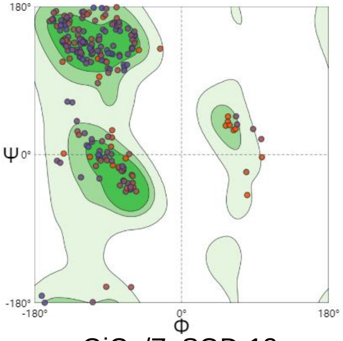

OjCu/ZnSOD-13

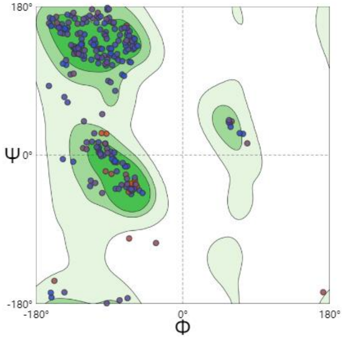

OjCu/ZnSOD-14

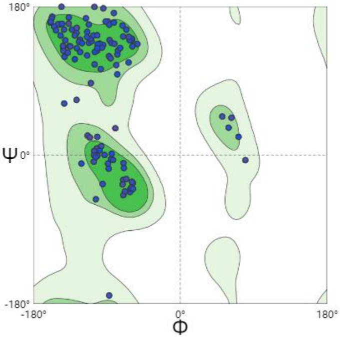

OjCu/ZnSOD-15

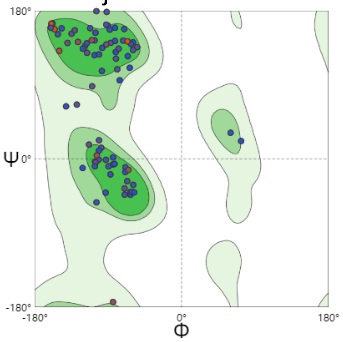

OjCu/ZnSOD-16

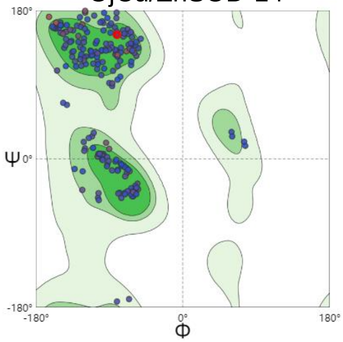

OjCu/ZnSOD-17

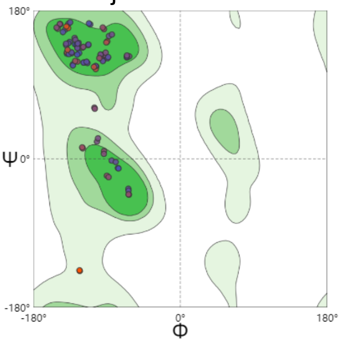

OjCu/ZnSOD-18

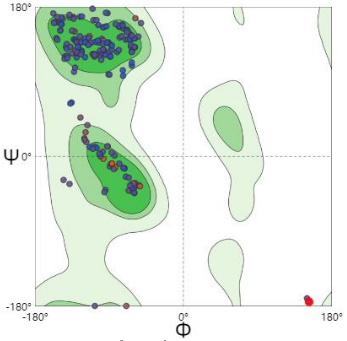

OjCu/ZnSOD-19

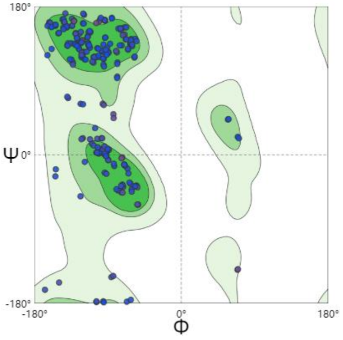

OjCu/ZnSOD-20

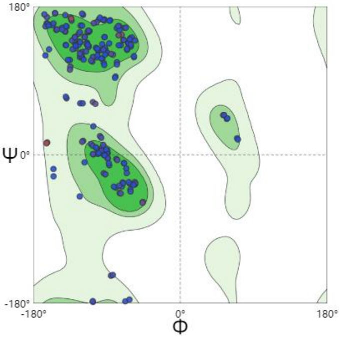

OjCu/ZnSOD-21

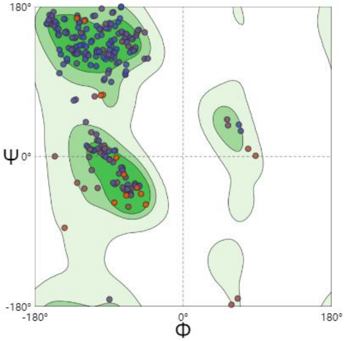

OjCu/ZnSOD-22

Supplementary Figure 3 Ramachandran plot of OjSOD proteins.

A

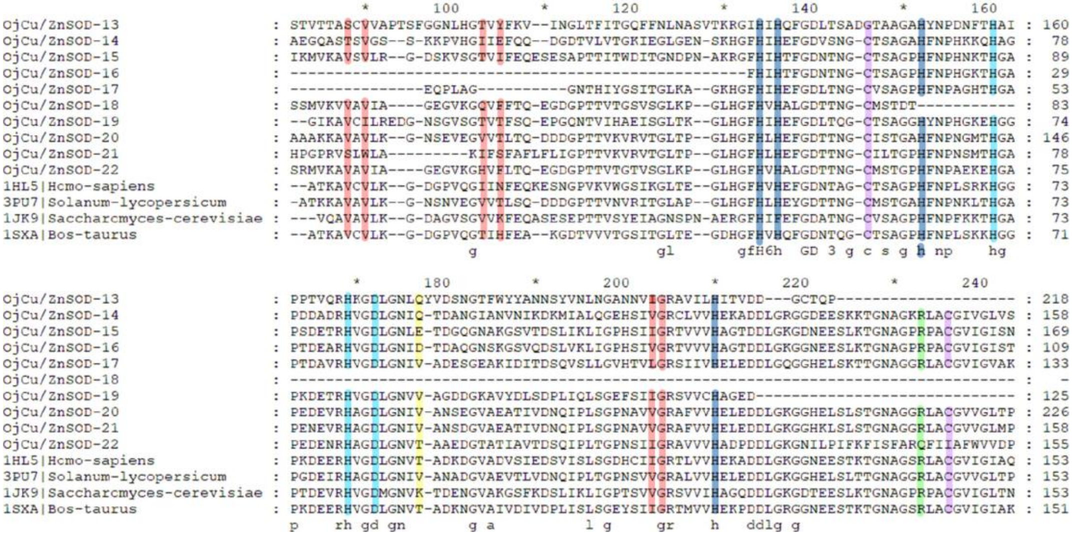
OjCu/ZnSOD-13
OjCu/ZnSOD-14
OjCu/ZnSOD-15
OjCu/ZnSOD-16
OjCu/ZnSOD-17
OjCu/ZnSOD-18
OjCu/ZnSOD-19
OjCu/ZnSOD-20
OjCu/ZnSOD-21
OjCu/ZnSOD-22
1HL5|Hcmo-sapiens
3PU7|Solanum-lycopersicum
1JK9|Saccharcmyces-cerevisiae
1SXA|Bos-taurus

B

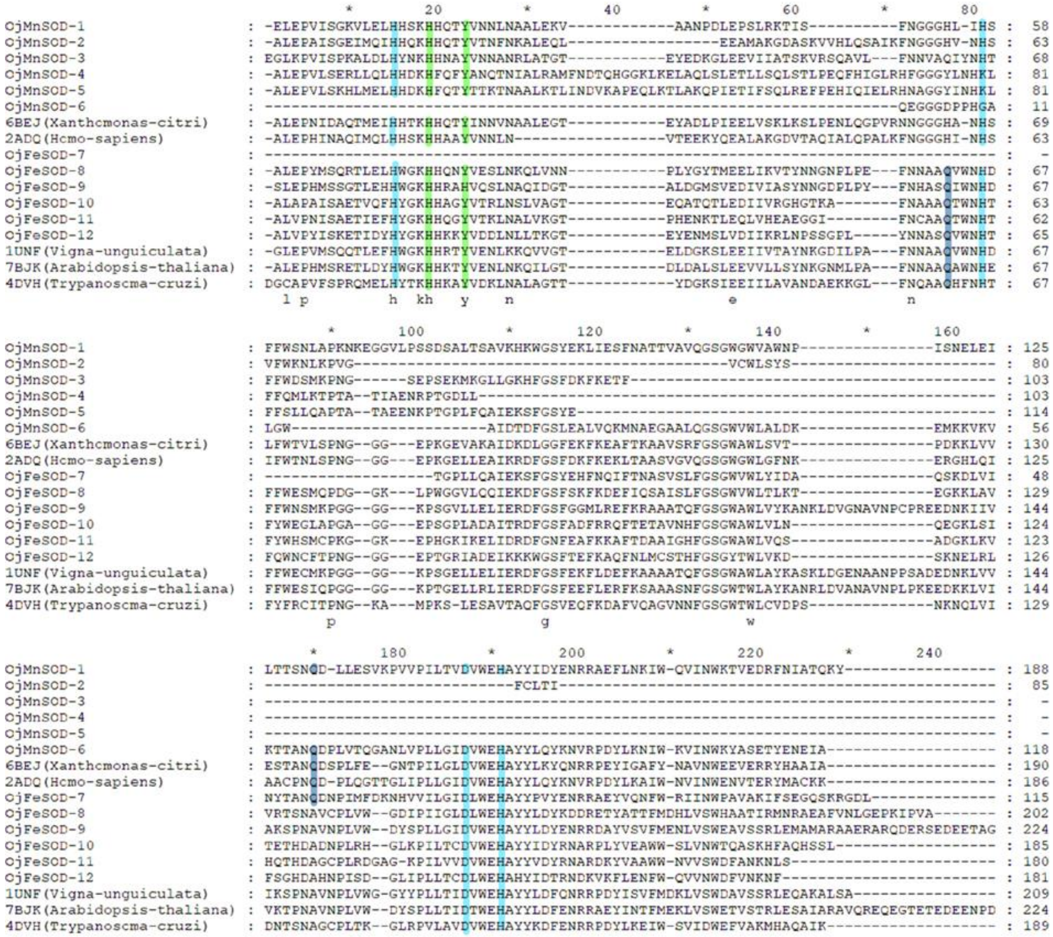
OjMnSOD-1
OjMnSOD-2
OjMnSOD-3
OjMnSOD-4
OjMnSOD-5
OjMnSOD-6
6BEJ(Xanthcmonas-citri)
2ADQ(Hcmo-sapiens)
OjFeSOD-7
OjFeSOD-8
OjFeSOD-9
OjFeSOD-10
OjFeSOD-11
OjFeSOD-12
1UNF(Vigna-unguiculata)
7BJK(Arabidopsis-thaliana)
4DVH(Trypanoscma-cruzi)

Supplementary Figure 4 Multiple sequence alignment of OjSODs. (A) Multiple sequence alignment of OjCu/ZnSODs with sequences from the same class for which the PDB structure was experimentally determined. Residues at the class E dimer interface were red; residues at the class P dimer interface were in yellow; residues involved in Cu ion binding were colored blue, while those involved in Zn ion binding were light blue. Dark blue circles indicated residues involved in the binding of both Cu and Zn ions. Two cysteine residues contributing to the formation of disulfide bonds were colored purple, and the residue responsible for attracting $O_2^{\cdot -}$ was marked in green. (B) Multiple sequence alignment of OjMnSODs OjFeSODs with sequences from the same class for which the PDB structure was experimentally determined. Residues involved in metal ion binding were light blue and residues involved in water molecule hydrogen binding were blue.

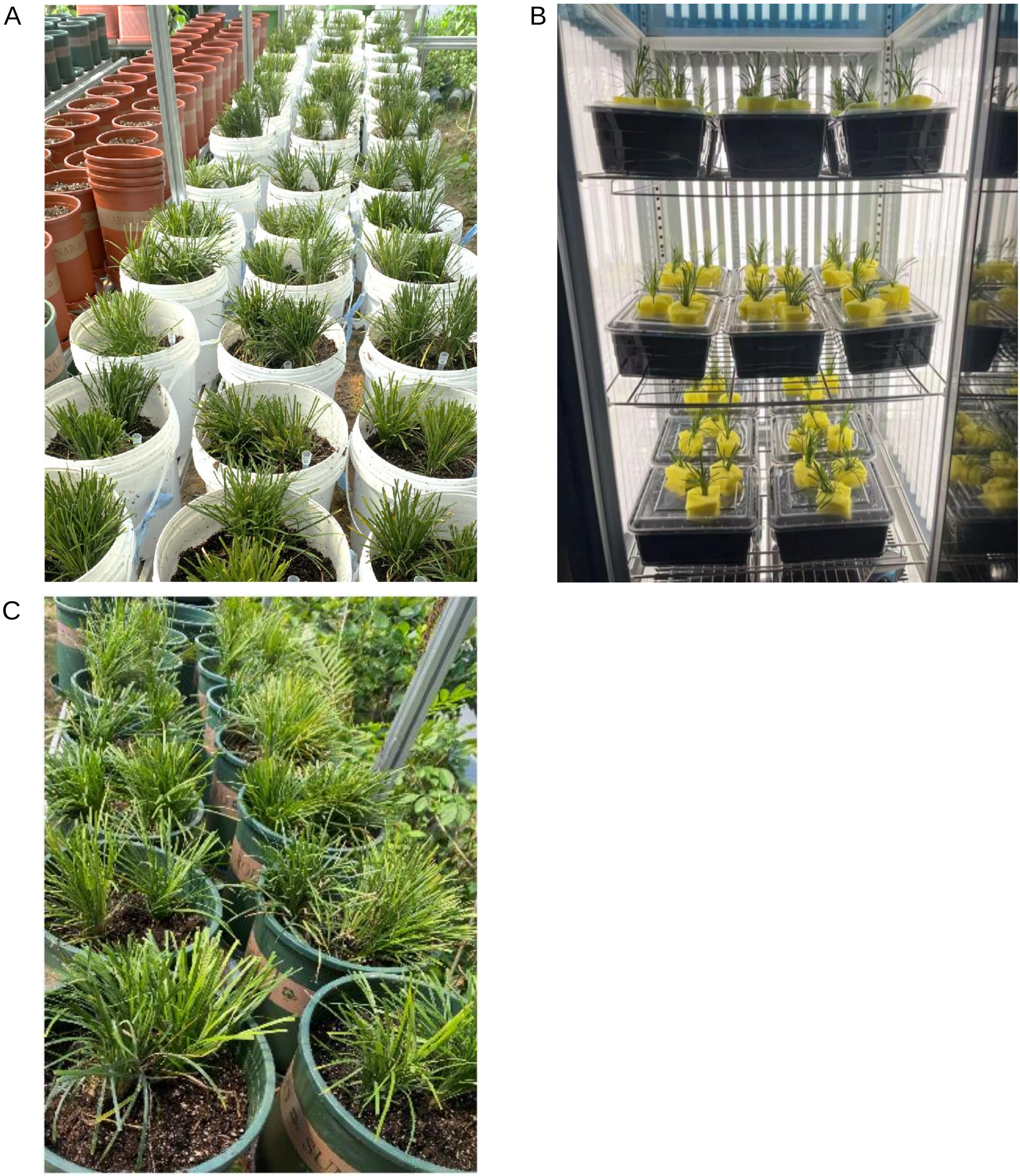
A
B
C

Supplementary Figure 5 The images of hydroponic and soil cultures. Different Cd stresses were performed on Zhe-Maidong by soil culture(A) and hydroponics(B). (C) Various heavy metal stress experiments were conducted on Zhe-Maidong, including Cu^{2+}, Fe^{2+}, Zn^{2+}, and Mn^{2+}.
